# Laser desorption/ionization-mass spectrometry for the analysis of interphases in lithium ion batteries

**DOI:** 10.1016/j.isci.2023.107517

**Published:** 2023-07-31

**Authors:** Valentin Göldner, Linda Quach, Egy Adhitama, Arne Behrens, Luisa Junk, Martin Winter, Tobias Placke, Frank Glorius, Uwe Karst

**Affiliations:** 1 Institute of Inorganic and Analytical Chemistry, University of Münster, Corrensstraße 48, 48149 Münster, Germany; 2 International Graduate School for Battery Chemistry, Characterization, Analysis, Recycling and Application (BACCARA), University of Münster, Corrensstraße 40, 48149 Münster, Germany; 3 Institute of Organic Chemistry, University of Münster, Corrensstraße 36, 48149 Münster, Germany; 4 MEET Battery Research Center, Institute of Physical Chemistry, University of Münster, Corrensstaße 46, 48149 Münster, Germany; 5 Bruker Daltonics GmbH & Co. KG, Fahrenheitstraße 4, 28359 Bremen, Germany; 6 Helmholtz Institute Münster, IEK-12, Forschungszentrum Jülich GmbH, Corrensstraße 46, 48149 Münster, Germany

**Keywords:** Organic chemistry, Electrochemical energy storage, Analytical Electrochemistry, Interfacial electrochemistry

## Abstract

Laser desorption/ionization-mass spectrometry (LDI-MS) is introduced as a complementary technique for the analysis of interphases formed at electrode|electrolyte interfaces in lithium ion batteries (LIBs). An understanding of these interphases is crucial for designing interphase-forming electrolyte formulations and increasing battery lifetime. Especially organic species are analyzed more effectively using LDI-MS than with established methodologies. The combination with trapped ion mobility spectrometry and tandem mass spectrometry yields additional structural information of interphase components. Furthermore, LDI-MS imaging reveals the lateral distribution of compounds on the electrode surface. Using the introduced methods, a deeper understanding of the mechanism of action of the established solid electrolyte interphase-forming electrolyte additive 3,4-dimethyloxazolidine-2,5-dione (Ala-*N*-CA) for silicon/graphite anodes is obtained, and active electrochemical transformation products are unambiguously identified. In the future, LDI-MS will help to provide a deeper understanding of interfacial processes in LIBs by using it in a multimodal approach with other surface analysis methods to obtain complementary information.

## Highlights

• Electrode surfaces were analyzed with laser desorption/ionization-mass spectrometry
• SEI components derived from film-forming electrolyte additives were characterized
• Enhanced tandem mass spectrometry provides further structural information
• Mass spectrometry imaging visualizes lateral distributions on electrode surfaces

## Graphical abstract

**Figure:**
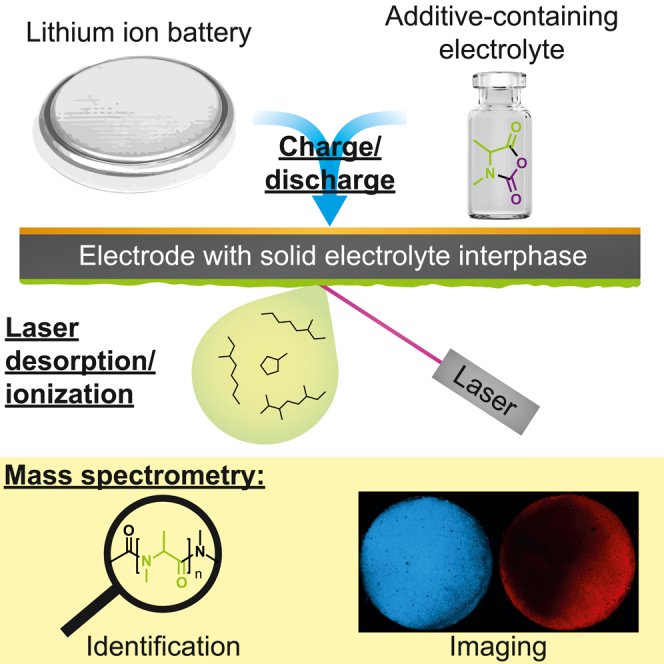

## Introduction

Over the past decade, the demand for emission-free transportation, particularly including battery electric vehicles, has increased substantially, and consequently, the need for efficient battery technologies with long lifetimes has grown.^1^ Due to their high-energy densities and outstanding power characteristics, lithium ion batteries (LIBs) have dominated the market so far.^2^ However, LIBs with increased energy density up to ≥750 Wh L^−1^ at cell level are urgently needed to fulfill future needs of the automotive sector in terms of longer driving ranges.^3^ To achieve these ambitious goals, a wide range of materials for both positive and negative electrodes is currently being studied.^4^ With respect to the negative electrode, silicon (Si) is one of the most promising materials for LIBs.^5^^,^^6^ This is mainly due to the fact that the theoretical specific capacity of Si (3579 mAh g^−1^, based on Li_15_Si_4_) is almost ten times higher than that of the state-of-the-art material, graphite (372 mAh g^−1^).^5^ Furthermore, Si is highly abundant in the earth’s crust and has a low-average discharge potential (0.4 V vs*.* Li|Li^+^), resulting in high-achievable cell voltages.^5^ Nevertheless, these advantages come with large challenges that hinder Si from broad commercial implementation in LIBs, including severe volume changes (≈300%) during charge and discharge processes.^5^ The continuous volume change leads to a dynamic surface and hinders the formation of an effective solid electrolyte interphase (SEI), thus continuously consumes the electrolyte and active lithium and, consequently, reduces the battery lifetime.^5^ One major strategy to address the challenges imposed by the use of Si electrodes is the formation of an optimized SEI, which can be achieved by the use of film-forming electrolyte additives that support the formation of a thin, flexible, and mechanically stable SEI.^7^^,^^8^

**Figure 1:**
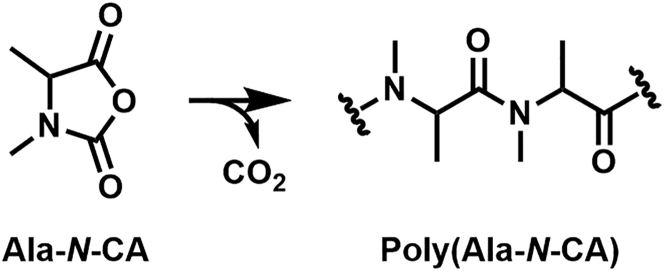
Proposed reaction for electrochemical oligomerization of 3,4-dimethyloxazolidine-2,5-dione (Ala-*N*-CA)^11^

SEI film-forming electrolyte additives are supposed to exhibit a low-molecular mass and should be ideally reductively decomposed in the first charge cycles at potentials higher than the electrolyte solvents.^9^ Besides the well-known additives vinylene carbonate (VC) and fluoroethylene carbonate (FEC),^10^ amino acid derived *N*-carboxyanhydrides (*N*-CAs)^11^ were identified as being beneficial for the LIB cell performance based on previous findings on *O*-carboxyanhydrides.^12^ This was attributed to the formation of oligopeptides, which efficiently protect the surface of the negative electrode after reduction of the *N*-CA additives at higher potentials than the electrolyte. 3,4-Dimethyloxazolidine-2,5-dione (Ala-*N*-CA), for example, was found to be reduced at 1.10 V vs. Li|Li^+^ whereas the baseline electrolyte was reduced at 0.70 V vs. Li|Li^+^.^11^ Utilizing high-performance liquid chromatography (HPLC) hyphenated with mass spectrometry (MS), small oligo(*N*-CA) structures were determined in the electrolyte, and a mechanism of action was proposed based on the detected species (see Figure 1).^11^ However, only structures that are soluble in the carbonate electrolyte are accessible for detection using HPLC/MS, while species immobilized on the electrode and incorporated in the SEI are not observed. X-ray photoelectron spectroscopy (XPS),^13^^,^^14^ Fourier-transform infrared (FTIR) spectroscopy^15^^,^^16^ and Raman spectroscopy^17^^,^^18^ are surface-sensitive techniques commonly used to characterize the chemical composition of the SEI.^19^ However, a full characterization of organic oligo- and polymers is not possible yet. This highlights the need for additional analytical techniques to study relevant processes and support the targeted design of film-forming additives.^9^^,^^20^

MS is a powerful tool for studying the chemical composition of SEI.^21^ Specifically, secondary ion mass spectrometry (SIMS) is well established for material analysis, yielding information about the inorganic and organic composition of surfaces.^22^ By scanning a sample with the primary ion beam, mass spectrometric images with lateral resolutions down to 5 nm can be generated.^19^ As secondary ions are almost exclusively formed from atoms and molecules in the uppermost surface layer, a uniquely high-depth resolution is achieved. Thus, molecular depth profiling and three-dimensional imaging with sub-micron resolution are possible.^23^^,^^24^^,^^25^ However, SIMS ionization generates not only quasi-molecular ions but also a large number of fragments, complicating the analysis of organic compounds with higher molecular mass, like oligo- and polymers. This is especially observed when polymers are not only present in the uppermost surface layer but are also entangled in the sample matrix.^22^ Thus, only small fragments of polymers are detected during SIMS analysis of SEI layers in many studies, and the polymers are described based on these fragments.^25^^,^^26^^,^^27^^,^^28^ A surface-sensitive ionization technique for mass spectrometry with a different ionization mechanism causing less fragmentation can therefore produce complementary information to SIMS.

Laser desorption/ionization (LDI) and matrix-assisted laser desorption/ionization (MALDI) are surface-sensitive ionization techniques that could fill this gap and complement information on organic polymers obtained by SIMS. Both LDI and MALDI are soft ionization techniques resulting in the formation of primarily singly charged, intact ions that can be used to identify even large organic species.^29^^,^^30^^,^^31^^,^^32^^,^^33^^,^^34^^,^^35^^,^^36^^,^^37^ MALDI is widely applied in biochemistry to study peptides,^38^ lipids,^39^^,^^40^ and metabolites.^41^ However, MALDI and LDI are also established as methods for the characterization of polymers^37^^,^^42^^,^^43^^,^^44^ and have recently been applied to study electrochemical side reactions on electrode surfaces.^45^ Characterization of SEI components using MALDI has also been reported.^46^^,^^47^^,^^48^^,^^49^^,^^50^ However, most examples suffer from poor mass resolution and can, therefore, not differentiate easily between polymeric SEI components and other species with equidistant signal distributions, like matrix clusters.^51^ Contrary to MALDI, LDI performs desorption and ionization without the addition of a matrix and is, therefore, less prone to error and more suited for routine analysis of electrode samples.

Isomeric species pose difficulties during MS analysis of complex samples as they are not differentiable using their mass-to-charge ratio (*m*/*z*). An additional separation is required, which is typically achieved by hyphenating chromatography and MS. However, chromatography is not applicable for surface analysis using (MA)LDI-MS, as ionization occurs directly from the sample surface. Therefore, a separation technique is required that can be implemented after ionization and before mass spectrometric detection. Ion mobility spectrometry (IMS) separates ions based on their mobility in the gas phase and, therefore, based on their size-to-charge ratio.^52^ Trapped ion mobility spectrometry (TIMS) is a form of IMS, where ions are transported by a steady carrier gas flow and are trapped by an opposing electrical field gradient. By stepwise decreasing the electrical field, the ions are serially eluted based on their mobility into an MS system where they are detected.^53^ The combination of (MA)LDI-TIMS-MS therefore allows the differentiation of isomeric species directly from electrode surfaces.

Apart from the mass spectrometric characterization of a variety of different compounds, (MA)LDI-MS can be applied as an imaging technique, depicting the lateral distribution of compounds on surfaces.^54^^,^^55^ While (MA)LDI-MS imaging (MSI) is well established in biomedical research for the analysis of biological tissues, the full potential of (MA)LDI-MSI is not yet employed in materials sciences. To the best of our knowledge, (MA)LDI-MSI of electrodes has only been reported once to visualize electrochemical side reactions on electrodes used for organic electrosynthesis.^45^ When studying SEI composition, the additional information obtained by MSI could further enhance the understanding of SEI formation processes and support the identification of weak spots during electrode manufacturing and cell assembly. Hence, LDI(-TIMS)-MS has great potential to support the understanding of SEI formation and rationalize electrolyte additive and interphase design (including SEI and cathode electrolyte interphase (CEI^56^)) in general.

## Results and discussion

### LDI-MS allows detection of Ala-*N*-CA-derived oligomers in the SEI

**Figure 2:**
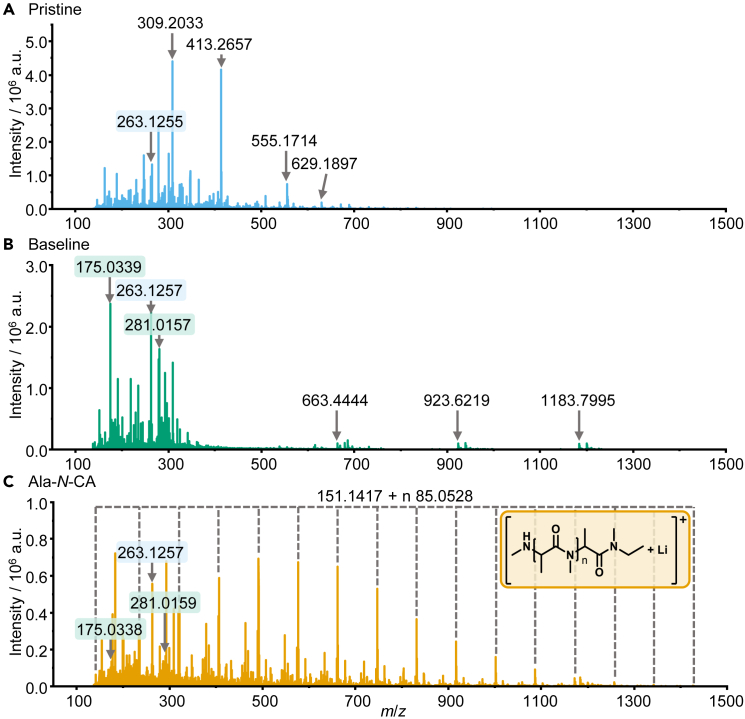
LDI-MS spectra obtained by analysis of of electrode surfaces (A) Pristine electrode. (B) Electrode cycled with the baseline electrolyte (LP57). (C) Electrode cycled with Ala-*N*-CA additive-containing electrolyte.

The applicability of LDI-MS for the analysis of SEI components derived from film-forming electrolyte additives is shown by the comparison of mass spectra obtained by the analysis of different electrodes. Pristine Si/graphite electrodes (Figure 2A) were compared to Si/graphite electrodes that were charged/discharged for three cycles at 0.1C in Si/graphite || Li-metal cells employing either the baseline electrolyte (LP57) (Figure 2B), which contained LiPF_6_ and the carbonate solvent but not the film-forming additive, or electrolyte with 2 wt.% of Ala-*N*-CA additive (Figure 2C, see Figure 1 for structure). Voltage profiles and Coulombic efficiencies of the three formation cycles are shown in the supplemental information (see Figures S1.1–S1.4) for the cells with and without Ala-*N*-CA additive. To preserve the native state of the SEI, all cells were disassembled and electrodes were mounted to sample holders in a glovebox without washing. Transfer to the mass spectrometer was realized under argon atmosphere. All analyses of cycled electrodes were performed in triplicate to ensure reproducibility. The data are conclusive for all electrodes of the triplicates (data shown in Figures S2.1 and S2.2). To identify SEI components, the differences between pristine electrodes, cycled electrodes, and cycled electrodes with Ala-*N*-CA have to be considered. Some signals, like *m*/*z* 263.1257, are observed on all analyzed electrodes and can therefore be considered to be derived from the electrode material. This indicates that LDI-MS desorbs and ionizes not only the top layer of the SEI but also analyzes the complete SEI layer and some of the supporting electrode in a single experiment. The desorption and ionization are likely induced by the absorption of the laser radiation by the dark surface of the graphite active material in the supporting electrode. Thus, the obtained chemical information can be assumed to represent the complete SEI. This ensures sufficient sensitivity of the method because more material is desorbed than if only the top layer is analyzed. On the other hand, depth information is lost and no statements about a possible layered structure of the SEI can be made using LDI-MS. Comparing the pristine electrode to the electrode cycled with baseline electrolyte, many differences are observed. The most intense species on cycled electrodes include *m*/*z* 175.0339 and *m*/*z* 281.0157. The assigned molecular formulae are C_4_H_9_LiO_5_P^+^ (Δ*m*/*z* = 2.0 ppm) and C_6_H_12_LiO_8_P_2_^+^ (Δ*m*/*z* = 1.9 ppm), respectively. Both compounds are, therefore, likely to be organophosphates, a class of compounds known to be formed as electrolyte degradation products in LIBs.^57^^,^^58^ This identification is supported by the mass spectrometric fragmentation patterns (data shown in Figures S3.1 and S3.2), but an unambiguous structural elucidation of these compounds is not possible due to the absence of distinct fragmentations characterizing the connectivity of the ethylene moieties. Additionally, three equidistant signals with a mass difference of 260.1775 Da (C_17_H_24_O_2_) are detected in the high-mass region of the electrode cycled with baseline electrolyte, which were not present on the pristine electrode. Similar signals are, however, also observed on uncycled electrodes after contact to the electrolyte, indicating that the compounds are contained in the electrolyte and are therefore not transformation products incorporated in the SEI (data shown in Figures S4.1 and S4.2).

To identify SEI components derived from Ala-*N*-CA, the results of the electrodes cycled with baseline electrolyte and additive-containing electrolyte are compared. While some signals, like *m*/*z* 175.0339 and *m*/*z* 281.0157, are detected with and without additives, a large quantity of additional signals is detected with Ala-*N*-CA. Several equidistant signals with a mass difference of 85.0528 Da (C_4_H_7_NO) are detected that show a typical intensity distribution related to an oligo- or polymer. The mass difference between the signals fits the expected repeating unit of poly(Ala-*N*-CA) that was proposed previously (Figure 2).^11^ Therefore, the incorporation of oligo(Ala-*N*-CA) in the SEI can be verified for the first time. Additionally, the end groups of the oligomer are identified, and the proposed structure of the formed oligomer is depicted in Figure 2C. It is likely that these electrochemically formed oligomers contribute greatly to the positive effect of Ala-*N*-CA on battery performance. On a more detailed look, several additional oligomer distributions are observed in the LDI-MS spectrum in Figure 2C. All of them share a common repeating unit with a mass of 85.0528 Da but are shifted by a constant value to the most intense oligomer signal discussed previously. The shifted oligomer signals are derived either from different ion species, like adducts with different metal ions or from structural alterations in the end groups. These oligomers with altered end groups are discussed in more detail in the supplemental information (see Figure S5.1) and can be attributed to hydrolytic oligomerization products and in-source fragments of larger oligomers. Hydrolytic oligomerization of Ala-*N*-CA was observed especially in electrodes that were immersed in Ala-*N*-CA-containing electrolyte for several days without cycling (see Figures S4.1 and S4.2).

### TIMS and MS/MS expand the scope of LDI-MS by providing further structural information

**Figure 3:**
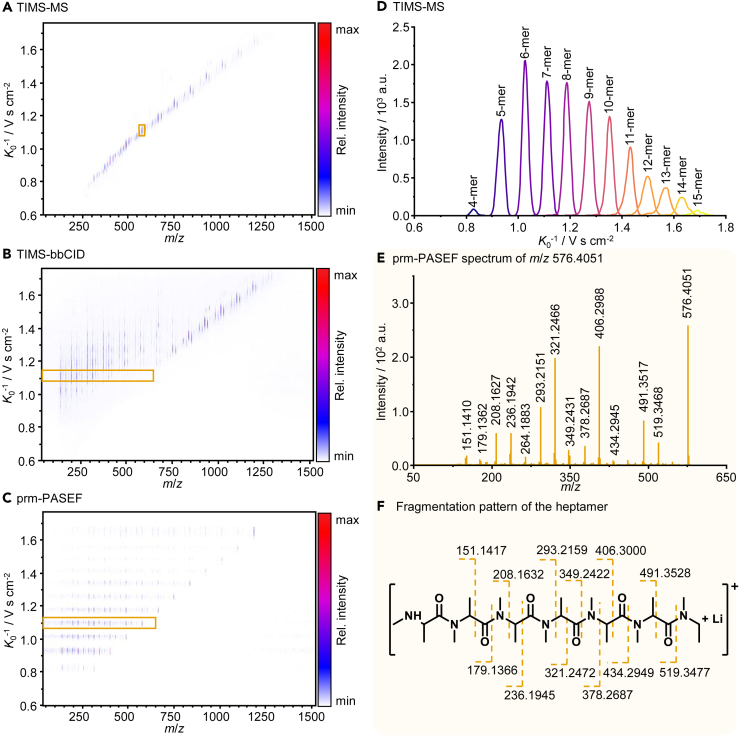
Results from LDI-TIMS-MS and LDI-TIMS-MS/MS analysis of Ala-*N*-CA-derived oligomers (A) Heatmap depicting the LDI-TIMS-MS data obtained from an electrode cycled with Ala-*N*-CA-containing electrolyte. (B) Heatmap obtained by TIMS-bbCID analysis of the same electrode. (C) Heatmap obtained by prm-PASEF analysis of electrochemically formed Ala-*N*-CA oligomers on the same electrode. (D) Extracted ion mobilograms from LDI-TIMS-MS analysis of differently sized electrochemical Ala-*N*-CA oligomers. (E) Fragment spectrum of the electrochemically formed heptamer (*m*/*z* 576.4051) extracted from the prm-PASEF dataset within the inverse reduced mobility range 1.069–1.113 V s cm^−2^. (F) Fragmentation pattern of the electrochemically formed Ala-*N*-CA heptamer.

The proposed structures based on the accurate masses should be validated by further experiments before possible reaction mechanisms are suggested. Due to the ionization of intact oligomers using LDI, a combination with further analysis techniques in the gas phase is possible. Therefore, TIMS was used to separate the ions based on their size-to-charge ratio before MS detection. This allows a differentiation between constitutional isomers which, in the case of oligomers, could be derived from different connectivity of the monomers. Given the molecular structure of *N*-CAs,^11^ the formation of linear oligomers is likely. For other film-forming additives like FEC, however, the formation of branched polymers has been observed and will affect the physical properties of the SEI due to differences in flexibility of branched and linear oligo- and polymers.^59^ Using LDI-TIMS-MS, branched and linear oligomers can be separated and the degree and type of cross-linking identified. Figure 3A depicts the LDI-TIMS-MS results of the cycled Si/graphite electrode using the Ala-*N*-CA-based electrolyte in a heatmap. By plotting the inverse reduced mobility (*K*_0_^−1^) against the *m*/*z*, a trajectory is formed, as increasing *m*/*z* correlates with an increasing *K*_0_^−1^. Only one mobility-resolved signal is observed for each *m*/*z* of the electrochemical oligomer, and all oligomer signals are separated in the mobility dimension. This can be observed more easily in the extracted ion mobilograms of the oligomers, depicted in Figure 3D. As expected, no branched oligomers are formed during electrochemical oligomerization, as they would likely be detected at different mobility values for the same *m*/*z*.

To validate the proposed structures, tandem mass spectrometry (MS/MS) was used to perform fragmentation experiments. Using LDI quadrupole time-of-flight instruments, only one compound can be fragmented per analyzed LDI spot, prolonging the required time for data acquisition. Additional implementation of the TIMS dimension, however, results in a temporal separation of ions with different mobilities. Therefore, multiple ions per LDI spot can be fragmented as long as they differ in mobility, speeding up the data acquisition.^60^ An untargeted approach for the utilization of this advantage is the combination of TIMS with broad-band collision-induced dissociation (bbCID). Here, all ions eluting from the TIMS tunnel are fragmented, and the fragments are subsequently detected via MS. The detected fragments can be assigned to their precursors as they share the same mobility value (i.e., are observed on a horizontal line in a TIMS-bbCID heatmap).^60^ This fragmentation approach is advantageous if the compounds of interest are unknown and as much information as possible should be acquired. However, multiple ions with similar mobilities may overlap when complex samples are analyzed, resulting in an elaborate data evaluation that is prone to misinterpretation. In the TIMS-bbCID heatmap of the cycled Ala-*N*-CA electrode (Figure 3B), such overlap in mobility is observed for different metal adducts and oligomers with different end groups. Additionally, the choice of fragmentation energy can pose difficulties when a large *m*/*z* range is analyzed. In the case of the cycled Ala-*N*-CA electrode (Figure 3B), the chosen collision energy is only adequate for oligomers of intermediate size (about *m*/*z* 600 – *m*/*z* 1000). Larger oligomers are not fragmented due to insufficient collision energy, while the energy is too high for smaller oligomers, fragmenting them to an extent where little to no structural information is obtained.

A targeted approach for TIMS-based MS/MS experiments is parallel reaction monitoring-parallel accumulation serial fragmentation (prm-PASEF). Here, signals are isolated using defined *m*/*z* and mobility windows and are then fragmented with specific fragmentation energy for each compound.^61^ So far, the method has mainly been applied in HPLC/MS^62^ and the application to (MA)LDI-MS is a more recent approach.^60^ Figure 3C depicts the heatmap obtained by prm-PASEF analysis of the cycled Ala-*N*-CA electrode with the electrochemical oligomers as target compounds. Isolation and fragmentation was performed based on the parameters presented in Table 1. Compared to the TIMS-bbCID heatmap, clean and easy to interpret spectra are obtained over the whole *m*/*z* and mobility range, proving prm-PASEF to be the ideal fragmentation method for the characterization of oligomers. The orange rectangle throughout Figures 3A–3C marks the signals derived from the electrochemically generated heptamer as an example. In Figure 3E, the fragment spectrum of the heptamer is depicted in a two-dimensional graph after extraction from the three-dimensional prm-PASEF data within the inverse reduced mobility range 1.069–1.113 Vs. cm^−2^. This MS/MS spectrum is compared to the spectrum obtained by LDI-TIMS-bbCID in the supplemental information (see Figure S6.1). Using the obtained data of all oligomers, the proposed linear structure and the end groups are validated. Figure 3F shows the proposed fragmentation pattern of the heptamer.

**Table 1:** Isolation width and fragmentation energy for prm-PASEF experiments based on the isolated mass-to-charge ratio (*m*/*z*)

	Type	Mass (*m*/*z*)	Width (*m*/*z*)	Collision energy (eV)
1	base	290.0	2.0	30.0
2	base	450.0	2.0	42.0
3	base	630.0	2.0	55.0
4	base	850.0	2.0	70.0
5	base	1000.0	2.0	81.0
6	base	1200.0	2.0	95.0

**Figure 4:**
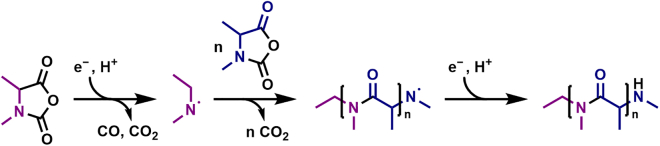
Proposed mechanism for the electrochemically initiated oligomerization of Ala-*N*-CA

Based on the validated structure, a mechanism is proposed for the electrochemical oligomerization (Figure 4). After initial electrochemical reduction, the loss of CO and CO_2_ leads to the formation of an amino radical which can undergo radical addition to another *N*-CA molecule, forming an amide in the process. Oligomerization and termination by reduction lead to the formation of the detected electrochemical Ala-*N*-CA oligomer. During electrochemical decomposition of *N*-CAs, both CO_2_ and CO were detected as products via gas analysis,^11^ which carbonate electrolytes are prone to in general as well,^63^ corroborating the proposed mechanism. A further discussion of the mechanism can be found in the supplemental information (see S7). Due to the knowledge obtained using LDI-TIMS-MS, the understanding of the underlying process during oligomer formation was improved, and previously proposed mechanisms^11^ were refined. The obtained information can help support targeted additive design by identifying and understanding alterations to the SEI.

### LDI-MSI generates images of oligomer distributions on electrode surfaces

**Figure 5:**
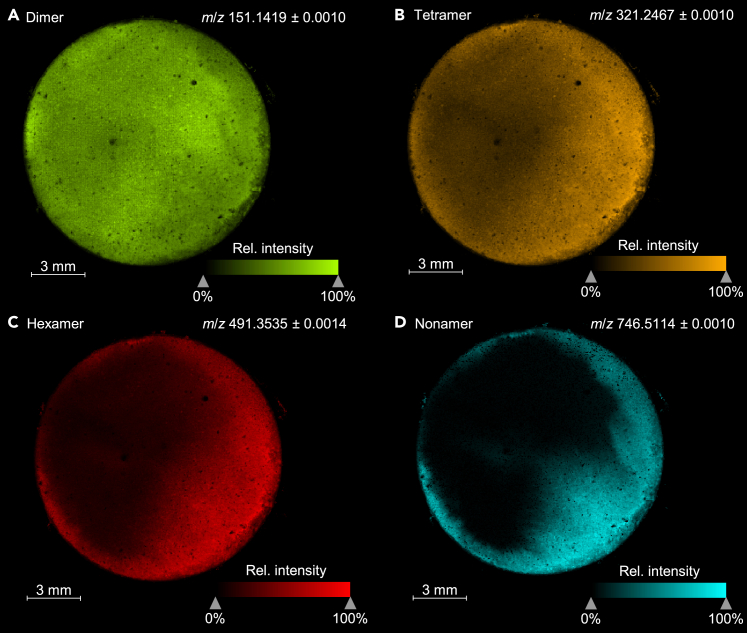
Mass spectrometric images of a Si/graphite electrode after three cycles with Ala-*N*-CA-containing electrolyte in Si/graphite || Li-metal cells Images of (A) the electrochemically formed dimer (*m*/*z* 151.1419, green), (B) the electrochemically formed tetramer (*m*/*z* 321.2467, orange), (C) the electrochemically formed hexamer (*m*/*z* 491.3535, red) and (D) the electrochemically formed nonamer (*m*/*z* 746.5114, turquoise).

In addition to the analysis of single spots on the electrode, the complete electrode surface was analyzed using LDI-MSI with a lateral resolution of 50 μm to ensure both sufficient spatial resolution and acceptable analysis time. The samples were charged/discharged for three cycles at 0.1C in Si/graphite || Li-metal cells before *post-mortem* analysis of the Si/graphite electrodes in triplicate. The resulting mass spectrometric images of one exemplary electrode are depicted in Figure 5. Figures 5A–5D show the lateral distribution of differently sized electrochemical oligomers derived from Ala-*N*-CA. The visualization shows a varying degree of oligomerization in different areas of the electrode. The dimer depicted in Figure 5A is distributed relatively homogenously, with slightly higher intensities in the center of the electrode. With increasing chain length (Figures 5B–5D), a more localized signal, focused toward the edges of the electrode, is observed. This trend is consistent in all triplicate samples (data shown in Figure S8.1). With regard to this observation, it is likely that oligomerization is initiated on the edges of the electrode where the larger oligomers are immediately immobilized and form an SEI layer. The solubility of small oligomers, especially the dimer, in the electrolyte is expected to be better than that of larger oligomers. This is supported by the fact that Ala-*N*-CA-derived dimers were detected in the electrolyte in previous studies.^11^ Consequently, small oligomers are more mobile and can diffuse before immobilization on the electrode surface, resulting in their more homogeneous distribution. A detailed discussion of the observed lateral distributions is provided in the supplemental information (see S8 and Figure S8.2). In coin cells, inhomogeneity has also been observed for the deposition of transition metals on negative electrodes which was attributed to different mechanical pressure inside the coin cell.^64^^,^^65^ Similar effects could possibly also influence the oligomerization of the studied Ala-*N*-CA additive and result in the observed oligomer distributions. However, further studies are required to understand the underlying processes in detail. Future work should study the phenomenon in pouch cell or cylindrical cell setup as these reflect commercially relevant battery applications better.^66^ The cell geometry is expected to have a great impact on oligomer immobilization patterns and, therefore, different oligomerization homogeneity might be observed. The use of pouch cells also allows to easily study the influence of mechanical pressure.^64^ Additionally, further additives with different moieties attached to the *N*-CA core structure should be investigated. Here, LDI-MS can be used to establish the homogeneity of the formed SEI as a new criterion during additive design.

### Conclusion

In conclusion, LDI-MS greatly enhances the identification of SEI components and is a valuable addition to the techniques already applied for SEI characterization. The obtained information is complementary to the results from other analysis techniques, like XPS, FTIR, and Raman spectroscopy, and enables the chemical characterization of crucial organic SEI components without the need for elaborate sample preparation. Compared to other surface-sensitive MS techniques, like SIMS, LDI-MS provides valuable additional information. The ionization of intact polymers and other organic molecules with a low amount of fragmentation in LDI-MS provides the opportunity for the combination with further gas-phase analysis techniques like TIMS and MS/MS. Thus, a deeper understanding of molecular structures can be obtained, including the connectivity and the end groups of oligo- and polymers. As LDI-MS does not only analyze the top surface layer, more material is desorbed and ionized, increasing the sensitivity of the method and ensuring that the data represents the chemical information of the complete SEI layer. Compared to MALDI, LDI provides several advantages when ionization of the analytes can be achieved without the application of a matrix. Most commonly used MALDI matrices are acidic and can initiate chemical alterations of the SEI components. Additionally, the matrix application process as well as possibly required washing steps can affect the structural integrity of the SEI. This does not only affect the resulting identification of SEI components but also their lateral distribution on the electrode and, therefore, the interpretation of mass spectrometric images. Using LDI, these effects are ruled out. Furthermore, LDI simplifies the data evaluation as matrix-derived signals in the lower mass region, which may overlap with signals derived from SEI components in MALDI-MS spectra, are not observed in LDI-MS analyses. In addition, no matrix clusters with equal mass spacing, which could be mistaken for oligo- and polymers, are observed in LDI. The use of TIMS as a further separation technique extends the applicability of LDI-MS even further. The obtained information about the mobility of the detected compounds helps with the differentiation of isomers like branched and linear oligomers. Moreover, the ion mobility separation enables faster characterization by using prm-PASEF for mass spectrometric fragmentation. Finally, LDI-MS cannot only be used for characterization but also enables the visualization of lateral distributions of different species on electrode surfaces using MSI. In this work, the flexible applications of LDI(-TIMS)-MS, including fragmentation and imaging experiments, were demonstrated for the analysis of the film-forming electrolyte additive Ala-*N*-CA. The derived oligomers were thoroughly characterized, and the lateral distribution on electrode surfaces was analyzed. The obtained data enhanced the understanding of the positive effect of Ala-*N*-CA on the performance of LIBs shown in previous work^11^ and the underlying processes. LDI-MS complements established analysis methods for electrode surfaces and addresses the gap for a soft ionization technique to study and characterize intact organic SEI components with MS.

### Limitations of the study

In this study, LDI-MS is discussed as a surface-sensitive technique for the characterization of organic SEI components in LIBs. The method is limited by the ionization process for which the sample surface needs to absorb radiation at the laser’s emission wavelength. Additionally, the complex composition of the analyzed SEI layer can cause ion suppression, possibly resulting in low-signal intensities for some compound classes. For imaging applications, the lateral resolution of LDI-MS is limited to a spot size of several micrometers and no-depth profiling is possible. In this work, LDI-MS was applied for *ex situ* characterization of battery electrodes. Especially for a real-time observation of SEI formation and for an evaluation of changes to the SEI during disassembly of LIBs, *in situ* and *in operando* analyses are beneficial. Thus, further improvement of LDI-MS instrumentation is required to enable this next step.

## STAR★Methods

### Key resources table

**Table:** 

REAGENT or RESOURCE	SOURCE	IDENTIFIER
**Chemicals, peptides, and recombinant proteins**		

3,4-dimethyloxazolidine-2,5-dione (Ala-*N*-CA)	Schmiegel et al. 2021^11^	N/A
Composite of ≈15 wt.% Si nanowires and ≈85 wt.% graphite	R&D material, not commercially available	N/A
Carbon black	Imerys Graphite & Carbon, Paris, France	Super C65
Sodium carboxymethyl cellulose	Dow Wolff Cellulosics, Bomlitz, Germany	Walocel CRT 2000 PPA12
Polyacrylic acid; average M_v_ 450,000	Sigma-Aldrich Chemie GmbH, Steinheim, Germany	Cat# 181285
1 m LiPF_6_ in 3:7 *w*/*w* ethylene carbonate (EC)/ethyl methyl carbonate (EMC)	Solvionic, Toulouse, France	N/A
Lithium metal foil; battery grade; purity ≥99.9%	China Energy Lithium (CEL Co.), Tianjin, China	N/A
Indium tin oxide-coated microscopic glass slides	Sigma-Aldrich Chemie GmbH, Steinheim, Germany	Cat# 576352
Super smooth conductive double sided carbon tape	Micro to Nano, Haarlem, The Netherlands	Cat# 15-000720

**Software and algorithms**		

timsControl	Bruker Daltonics GmbH & Co. KG, Bremen, Germany	Version 3.0, prototype
timsControl	Bruker Daltonics GmbH & Co. KG, Bremen, Germany	Version 3.1
flexImaging	Bruker Daltonics GmbH & Co. KG, Bremen, Germany	Version 7.0
DataAnalysis	Bruker Daltonics GmbH & Co. KG, Bremen, Germany	Version 6.0
SCiLS Lab	Bruker Daltonics GmbH & Co. KG, Bremen, Germany	Version 2023a

### Resource availability

#### Lead contact

Further information and requests for resources and reagents should be directed to and will be fulfilled by the lead contact, Uwe Karst (uk@uni-muenster.de).

#### Materials availability

This study did not generate new unique reagents.

### Method details

#### Preparation of 3,4-dimethyloxazolidine-2,5-dione

3,4-Dimethyloxazolidine-2,5-dione (Ala-*N*-CA) was synthesized following a modified literature procedure from the according amino acid l-alanine.^67^ Detailed synthesis information is provided in the supplemental information (see S9). NMR spectra of the synthesis intermediate *N*-(*tert*-butoxycarbonyl)-*N*-methylalanine and the final product are provided in Figures S9.1–S9.3.

#### Electrode preparation

The Si/graphite electrodes comprised 85 wt.% of active materials (a composite of ≈15 wt.% Si nanowires and ≈85 wt.% graphite) (BET surface area: 16 m^2^ g^−1^; d50: 17.7 μm, d90: 21.5 μm) with a theoretical specific capacity of ≈713 mAh g^−1^, 5 wt.% carbon black as conductive agent (Super C65, Imerys Graphite & Carbon, Paris, France), 7.7 wt.% sodium carboxymethyl cellulose (Na-CMC, Walocel CRT 2000 PPA12, Dow Wolff Cellulosics, Bomlitz, Germany) and 2.3 wt.% polyacrylic acid (PAA, average M_v_ 450,000, Sigma-Aldrich Chemie GmbH, Steinheim, Germany) as binders. The paste was prepared with deionized water as solvent. In the beginning of paste preparation, 1.2 wt.% of lithium hydroxide (LiOH, 98%, Fisher Chemical, Schwerte, Germany) were dissolved with the binders in a planetary centrifugal mixer (20 min, 1700 rpm, ARM-310CE, Thinky Corporation, Laguna Hills, USA). Afterwards, conductive agent, active material and deionized water were added and homogenized again with the same rotation speed. The anode paste was coated on smooth copper foil (20 μm, Nippon Steel, Tokyo, Japan) with a blade gap of 50 μm. After pre-drying at 70°C in an atmospheric oven for 2 h, the sheets were dried in an oven at 90°C for 8 h under reduced pressure. The electrodes were then punched out in discs with a diameter of 14 mm. The average active mass loading of the anodes was ≈1.24 ± 0.03 mg cm^−2^, resulting in an areal capacity of ≈0.88 ± 0.02 mAh cm^−2^ assuming a practical capacity of the Si/graphite composite of ≈700 mAh g^−1^.

#### Cell assembly and electrochemical characterization

Electrochemical experiments were carried out in a glovebox (O_2_/H_2_O level < 0.5 ppm) in two-electrode coin cells (CR2032, Hohsen Corporation, Osaka, Japan).^66^ Si/graphite (Ø=14 mm) composite electrode disks were used as a positive electrode and Li-metal as a negative electrode (Ø=15 mm, lithium metal foil, 500 μm in thickness; battery grade; purity ≥99.9%, China Energy Lithium (CEL Co.), Tianjin, China). As a separator, FS2190 separators (Ø=16 mm, 2 layers, Freudenberg, Weinheim, Germany) were soaked in 100 μL of the electrolyte LP57 (1 m LiPF_6_ in 3:7 *w*/*w* ethylene carbonate (EC)/ethyl methyl carbonate (EMC), Solvionic, Toulouse, France) with and without 2 wt.% of Ala-*N*-CA. To ensure reproducibility, the results of three cells per setup were used. Constant current followed by constant voltage (CCCV) charge-discharge cycling was performed on a Maccor Series 4000 battery tester (Maccor, Inc., Leicestershire, United Kingdom) at 20°C. Cells were then cycled for three cycles at 0.1C (1C was defined as 700 mA g^−1^) within the voltage range of 0.05 – 1.50 V for SEI formation.

#### Sample preparation

For *post*-*mortem* analysis, cells were disassembled in a glovebox (O_2_/H_2_O level < 0.5 ppm) in the discharged state at 1.5 V following the third cycle. It can be expected that the lithiation state of the negative electrode will not affect the detected species to a great extent because the chemical composition of the SEI is expected to be comparable in both states. However, a safe disassembly, analysis and disposal is easier to realize for delithiated negative electrodes. The unwashed Si/graphite electrodes were attached to indium tin oxide (ITO)-coated microscopic glass slides (70-100 Ω sq^−1^, Sigma-Aldrich Chemie GmbH, Steinheim, Germany) in the glovebox using conductive double-sided adhesive carbon tape (Micro to Nano, Haarlem, The Netherlands). The sample slides were mounted to the sample carrier in an Ar-flushed airlock and inserted in the evacuated (≈2.6 mbar) LDI source of the mass spectrometer for analysis without contact to ambient air.

#### Mass spectrometry

A timsTOF fleX (Bruker Daltonics GmbH & Co. KG, Bremen, Germany) mass spectrometer equipped with a 10 kHz frequency tripled Nd:YAG laser (355 nm) was used for LDI-MS, LDI-MSI, LDI-TIMS-MS and LDI-TIMS-MS/MS analyses. Single LDI-MS, LDI-TIMS-MS and LDI-TIMS-MS/MS spectra were recorded by summation of 17 spots distributed over the surface of the complete analyzed electrode. The mass spectrometer was controlled by timsControl 3.0 (Bruker Daltonics GmbH & Co. KG, Bremen, Germany). For LDI-prm-PASEF experiments, a prototype software version of timsControl 3.0 (Bruker Daltonics GmbH & Co. KG, Bremen, Germany) was used. LDI-MSI experiments were performed using flexImaging 7.0 software and timsControl 3.1. LDI-MS, LDI-TIMS-MS and LDI-TIMS-MS/MS data were evaluated using DataAnalysis 6.0 software (Bruker Daltonics GmbH & Co. KG, Bremen, Germany). LDI-MSI data was visualized using SCiLS Lab, Version 2023a (Bruker Daltonics GmbH & Co. KG, Bremen, Germany). Intensities from LDI-MSI data were normalized using root mean square normalization in SCiLS Lab, which gave the best normalization results for this application.^68^

##### LDI ionization was performed using the following parameters

LDI-MS, LDI-TIMS-MS, LDI-TIMS-MS/MS:

Laser application: MS dried droplet; power boost: 0.0%; smart beam: M5 defocus; beam scan: on; beam scan size X: 25 μm; beam scan size Y: 25 μm; laser energy: 1%; laser frequency: 5000 Hz; shots per spot: 500; movement on sample spot: random, partial sample; 10 shots per raster spot; limit diameter to 2000 μm.

LDI-MSI:

Laser application: Imaging 50 μm; power boost: 0.0%; smart beam: M5 small; beam scan: on; beam scan size X: 11 μm; beam scan size Y: 11 μm; laser energy: 65%; laser frequency: 5000 Hz; shots per spot: 200; movement on sample spot: off.

For LDI-MSI analyses, proper focus of the laser was ensured by adjusting the sample height using stainless steel spacers and fine tuning the z-position using the instruments MALDI stage.

##### MS detection was performed using the following parameters

Mode: MALDI(+) (without application of matrix); *m*/*z* 50-1500; funnel 1 RF: 350.0 Vpp; funnel 2 RF: 350 Vpp; isCID energy: 0.0 eV; multipole RF: 600.0 Vpp; deflection delta: 30 V; MALDI plate offset: 30 V quadrupole ion energy: 10.0 eV; quadrupole low mass: *m*/*z* 40; collision energy: 10.0 eV; collision RF: 1000 Vpp; transfer time: 80 μs; pre pulse storage: 10 μs.

##### TIMS-MS detection was performed using the following parameters

Mode: MALDI(+) (without application of matrix); *m*/*z* 50-1500; funnel 1 RF: 400.0 Vpp; funnel 2 RF: 350 Vpp; isCID energy: 0.0 eV; multipole RF: 600.0 Vpp; deflection delta: 30 V; MALDI plate offset: 30 V quadrupole ion energy: 10.0 eV; quadrupole low mass: *m*/*z* 40; collision energy: 10.0 eV; collision RF: 1000 Vpp; transfer time:70 μs; pre pulse storage: 5 μs.

TIMS mode: custom; 1/*K*_0_ start: 0.60 V s cm^−2^; 1/*K*_0_ end: 1.80 V s cm^−2^; ramp time 50.0 ms; accumulation time: 40.0 ms; duty cycle: 80.0%; ramp rate: 17.84 Hz; offsets: Δ1: −20.0 V, Δ2: −160.0 V, Δ3: 110.0 V, Δ4: 80.0 V, Δ5: 0.0 V, Δ6: 80.0 V, collision cell in: 300.0 V.

TIMS-bbCID experiments were performed at a collision energy of 70 eV.

The collision energy and isolation width for prm-PASEF experiments were adjusted automatically based on the isolated *m*/*z* according to Table 1.

The *m*/*z* and the mobility region for isolation of oligomers during prm-PASEF experiments were defined based on LDI-TIMS-MS data.

## Data Availability

• The data reported in this paper will be shared by the lead contact upon request. • This paper does not report original code. • Any additional information required to reanalyze the data reported in this paper is available from the lead contact upon request.
