# Supplementary material for: Laser desorption/ionization-mass spectrometry for the analysis of interphases in lithium ion batteries
Source: iScience. 2023 Jul 31;26(9):107517. doi: 10.1016/j.isci.2023.107517 (PMC10448071; doi:10.1016/j.isci.2023.107517)
Supplement: Document S1. Figures S1–S9 [file mmc1.pdf]

iScience, Volume *26*

Supplemental information

Laser desorption/ionization-mass spectrometry for the analysis of interphases in lithium ion batteries

Valentin Göldner, Linda Quach, Egy Adhitama, Arne Behrens, Luisa Junk, Martin Winter, Tobias Placke, Frank Glorius, and Uwe Karst

S1 Electrochemical data

The voltage profiles of the first three charge/discharge cycles of representative Si/graphite || Li-metal cells at 0.1C employing either the baseline electrolyte (LP57) or electrolyte with 2 wt.% of 3,4-dimethyloxazolidine-2,5-dione (Ala-*N*-CA) additive are depicted in figure S1.1 and figure S1.2, respectively. Figures S1.3 and S1.4 depict the mean coulombic efficiencies and standard deviations of the first three charge/discharge cycles of the triplicate cells with and without additive, respectively.

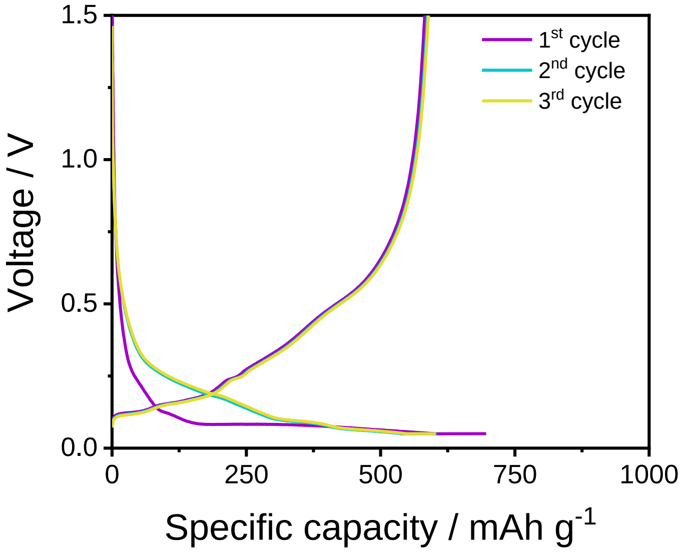

Figure S1.1. Voltage profiles of the first three charge/discharge cycles of a representative Si/graphite || Li-metal cell at 0.1C employing the baseline electrolyte LP57.

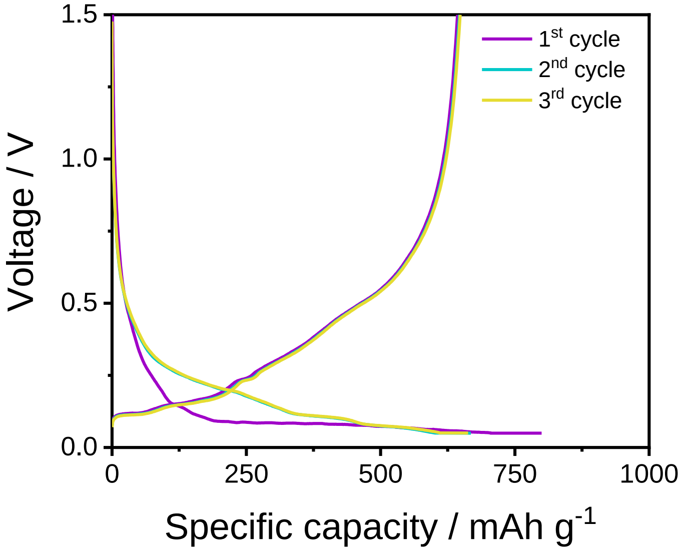

Figure S1.2. Voltage profiles of the first three charge/discharge cycles of a representative Si/graphite || Li-metal cell at 0.1C employing electrolyte containing 2 wt.% Ala-*N*-CA.

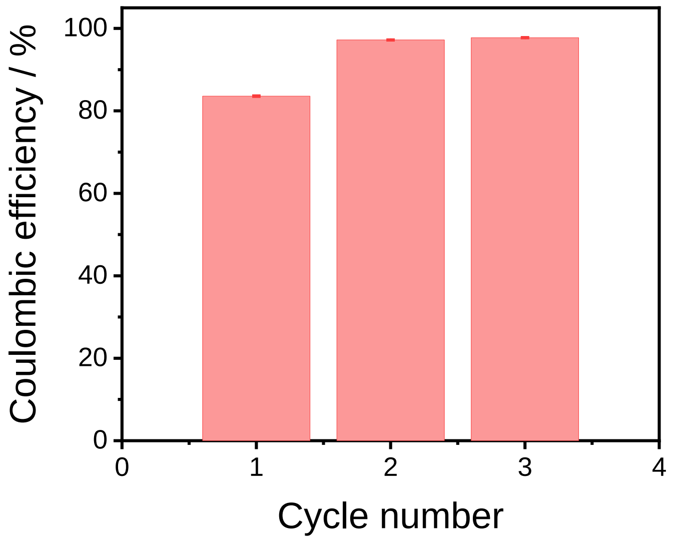

Figure S1.3. Mean Coulombic efficiencies of the first three charge/discharge cycles of triplicate Si/graphite || Li-metal cells at 0.1C employing the baseline electrolyte LP57. The error bars show standard deviation of a triplicate analysis

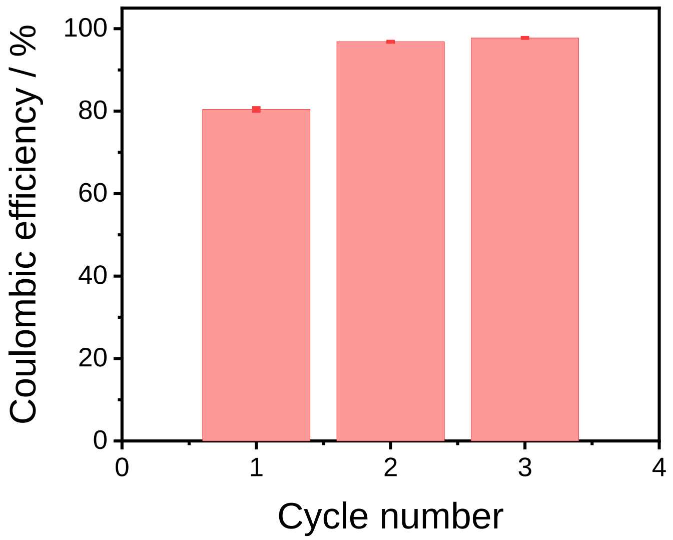

Figure S1.4. Mean Coulombic efficiencies of the first three charge/discharge cycles of triplicate Si/graphite || Li-metal cells at 0.1C employing electrolyte containing 2 wt.% Ala-*N*-CA. The error bars show standard deviation of a triplicate analysis

S2 Triplicate LDI-MS analysis

All cycled electrodes in this study were analyzed in triplicates. The data for one electrode of each set is shown in the main manuscript. Figures S2.1 and S2.2 depict the results of all samples from triplicate analysis of cells cycled with baseline electrolyte (LP57) and Ala-*N*-CA-containing electrolyte, respectively. Although the absolute intensities of the detected signals vary between samples, the signals discussed in the manuscript are detected on all analyzed electrodes. The difference in absolute signal intensities can be due to the ionization process of LDI which can be affected by sample height affecting the laser focus or by ion suppression.

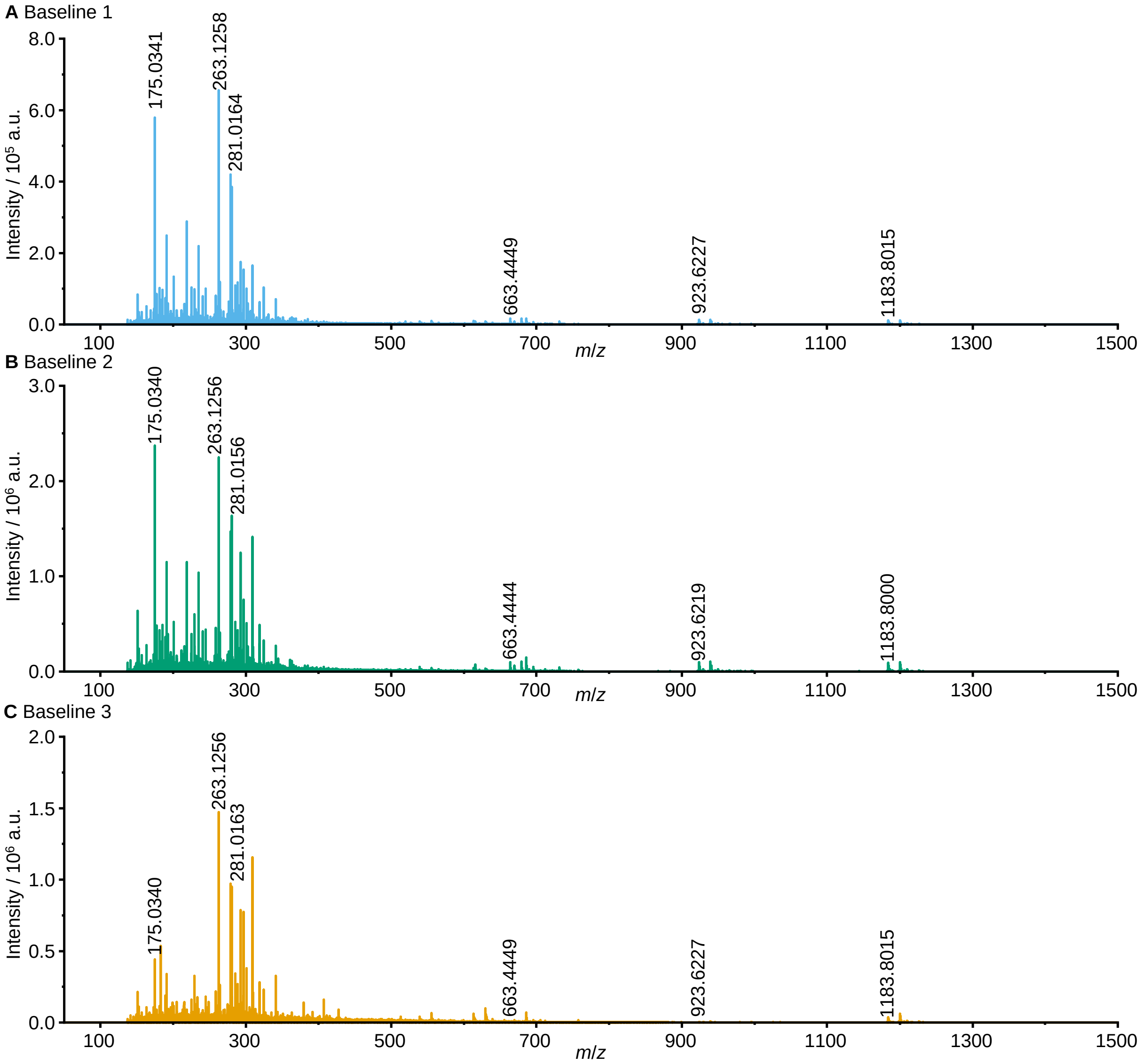

Figure S2.1. LDI-MS spectra of three electrodes cycled for three cycles with baseline electrolyte (LP57).

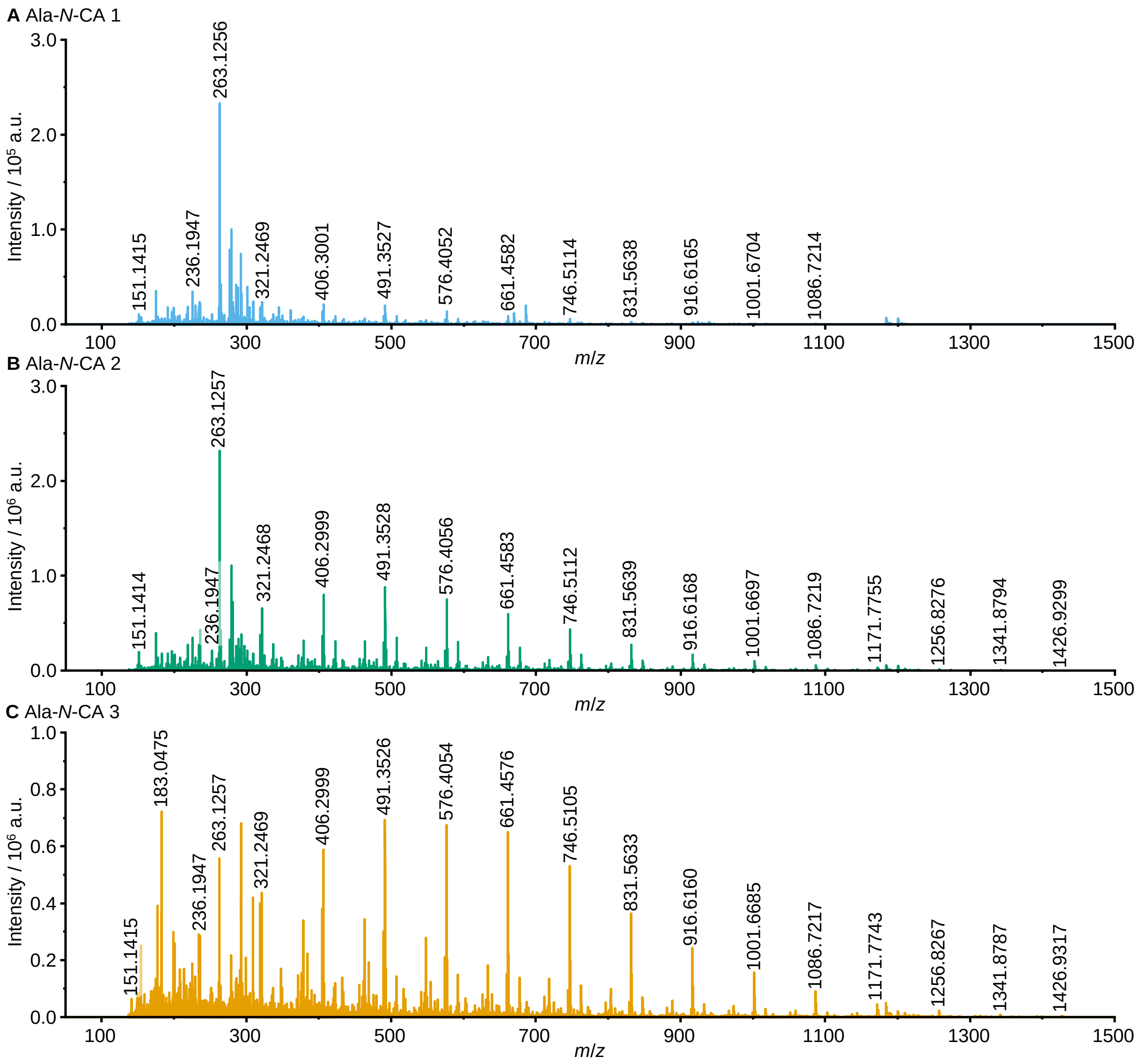

Figure S2.2. LDI-MS spectra of three electrodes cycled for three cycles with Ala-*N*-CA-containing electrolyte.

S3 LDI-MS/MS of detected organophosphates

Signals with *m*/*z* 175.0339 and *m*/*z* 281.0157 were detected both on cycled electrodes with baseline electrolyte and Ala-*N*-CA-containing electrolyte. The assigned molecular formulae based on the exact mass are $C_4H_9LiO_5P^+$ and $C_6H_{12}LiO_8P_2^+$, respectively. The molecular formulae may correspond to organophosphates, which are known electrolyte degradation products in LIBs.[1,2] To verify this assignment, MS/MS fragmentation of both compounds was performed. The resulting LDI-MS/MS spectra and fragmentation patterns for *m*/*z* 175.0345 and *m*/*z* 281.0165 are shown in figure S3.1 and S3.2, respectively. The depicted structural formulae are proposed structures among different possible isomers. For an unambiguous assignment of the structures, additional experiments are required.

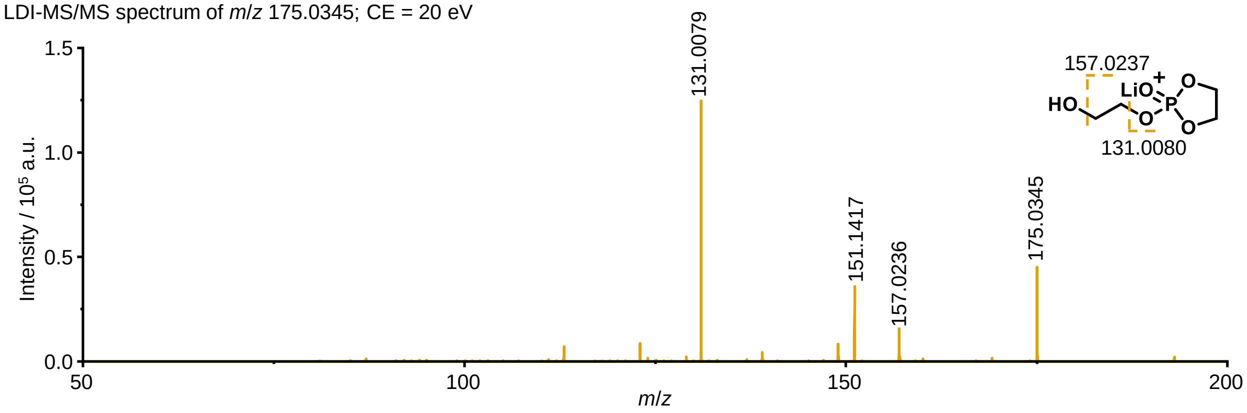

Figure S3.1. LDI-MS/MS spectrum and proposed fragmentation pattern of *m*/*z* 175.0345 at a collision energy of 20 eV. The fragmentation pattern includes the expected exact *m*/*z* values of the assigned fragments.

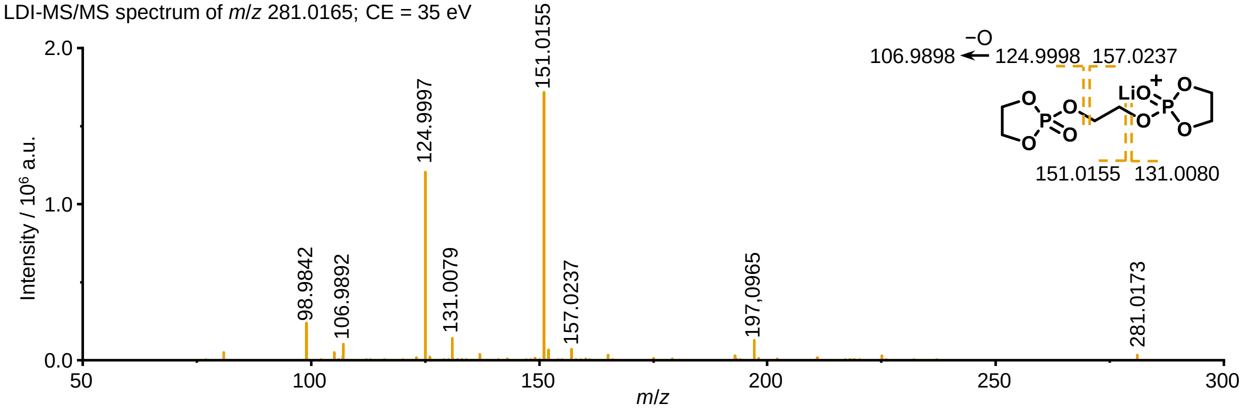

Figure S3.2. LDI-MS/MS spectrum and proposed fragmentation pattern of *m*/*z* 281.0165 at a collision energy of 35 eV. The fragmentation pattern includes the expected exact *m*/*z* values of the assigned fragments.

S4 LDI-MS analysis of uncycled electrodes

In addition to pristine and cycled Si/graphite electrodes, uncycled electrodes were analyzed. The electrodes were assembled in Li-metal coin cells containing 2 wt.% Ala-*N*-CA in the electrolyte. Figure S4.1 depicts the resulting LDI-MS spectrum after two days in the uncycled coin cell. The LDI-MS spectrum after 37 days in the uncycled coin cell is shown in figure S4.2. After long-time contact with Ala-*N*-CA-containing electrolyte, hydrolytic degradation of Ala-*N*-CA causes the formation of hydrolytic Ala-*N*-CA oligomers, which are observed in figure S4.2. During short-time contact (figure S4.1), only small hydrolytic oligomers are detected.

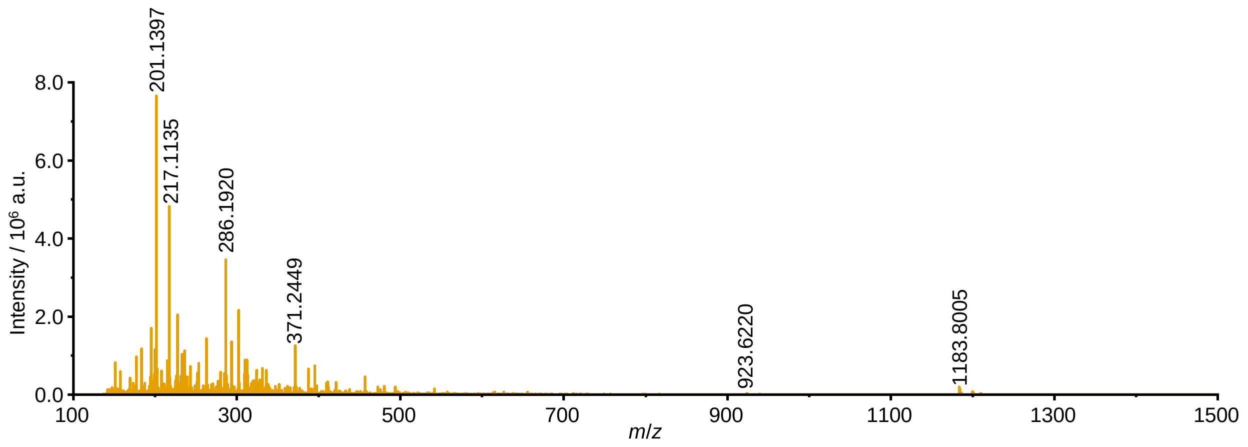

Figure S4.1. LDI-MS spectrum of an electrode after two days in an uncycled Li-metal coin cell containing 2 wt.% Ala-*N*-CA in the electrolyte.

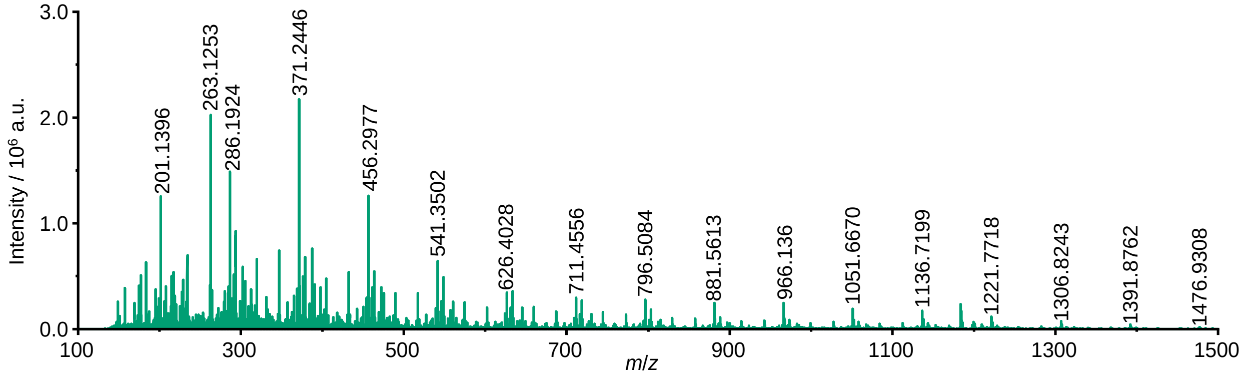

Figure S4.2. LDI-MS spectrum of an electrode after 37 days in an uncycled Li-metal coin cell containing 2 wt.% Ala-*N*-CA in the electrolyte.

S5 Additional observed Ala-*N*-CA oligomers

In the LDI-MS spectrum obtained by the analysis of an electrode cycled with Ala-*N*-CA-containing electrolyte, multiple signals with a common repeating mass unit of 85.0528 Da are detected. The signals are shifted by a constant value to the most intense oligomer signals discussed in the main manuscript. A zoomed region of the mass spectrum, depicting the signal of the electrochemically formed hexamer and all surrounding signals, is shown in figure S5.1. The shifted oligomer signals are derived either from different ion species like adducts with different metal ions or from structural alterations in the end groups. The signals with *m*/*z* 491.3526 ($[M+Li]^+$) and *m*/*z* 507.3261 ($[M+Na]^+$) can be attributed to the electrochemical Ala-*N*-CA oligomer discussed in the main manuscript. These signals are only detected on electrochemically cycled electrodes while all other oligomer signals are also detected on uncycled electrodes that were in contact with the additive-containing electrolyte for a longer period of time (see figure S4.2). Therefore, all further signals are assigned to be either hydrolytic oligomerization products of Ala-*N*-CA or in-source fragments of larger oligomers. The signal with *m*/*z* 456.2975 ($[M+Li]^+$) likely corresponds to an oligomer formed by hydrolysis while the signals with *m*/*z* 463.3213 ($[M+Li]^+$), *m*/*z* 469.3297 ($[M-H+2Li]^+$), *m*/*z* 479.2952 ($[M+Na]^+$) and *m*/*z* 489.3376 ($[M+Li]^+$) might correspond to in-source fragments of larger oligomers. Possible structures of the different oligomers are depicted in figure S5.1. The proposed structures should, however, be validated by further experiments.

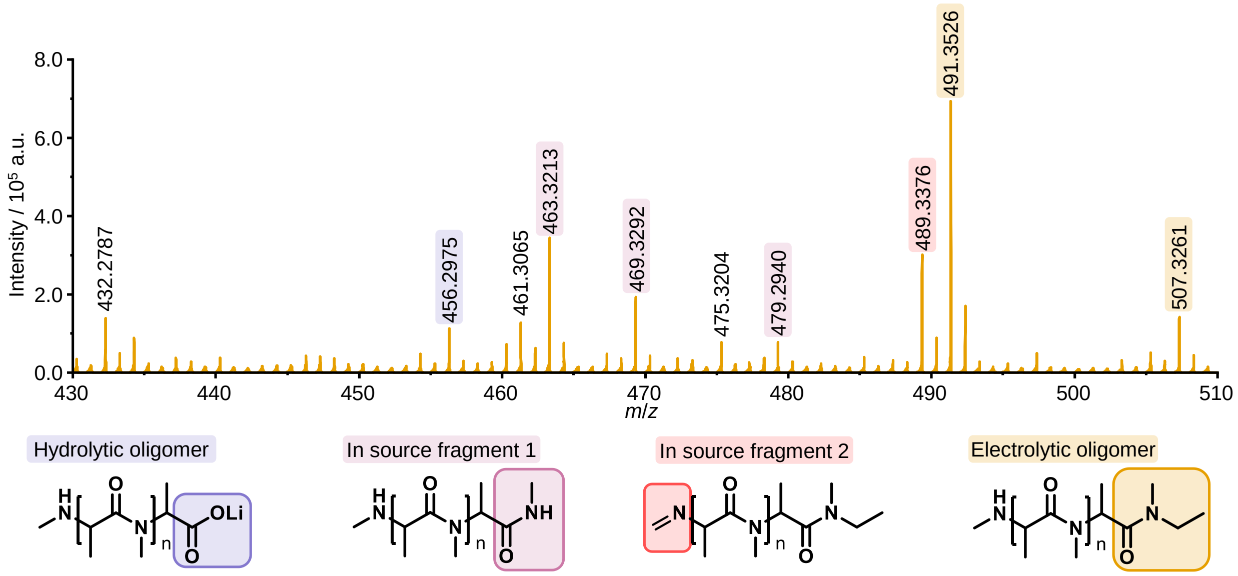

Figure S5.1. LDI-MS spectrum obtained by the analysis of an electrode cycled with Ala-*N*-CA-containing electrolyte. Zoomed region between *m*/*z* 430 and *m*/*z* 510 depicting the different metal adducts, oligomer species and in-source fragments derived from Ala-*N*-CA. The general structures of the detected oligomer species are depicted below the spectrum.

S6 Comparison between LDI-TIMS-bbCID and LDI-prm-PASEF

Comparing the extracted LDI-TIMS-bbCID and LDI-prm-PASEF data for the fragmentation of the electrochemically formed heptamer (*m/z* 576.4051) in the mobility range 1.069-1.113 V s cm^{-2}, the advantages of prm-PASEF over TIMS-bbCID can be visualized. Figure S6.1A shows the MS/MS spectrum of the heptamer acquired using TIMS-bbCID. Chimera fragment spectra are observed due to different oligomer signals overlapping in the mobility dimension (e.g., electrolytic oligomer and in-source fragment 2). Additionally, no precursor is detected because the applied collision energy is too high to preserve precursor ions. Figure S6.1B, on the other hand, shows a clear MS/MS spectrum for the hexamer with easily assignable fragment signals obtained by the use of prm-PASEF.

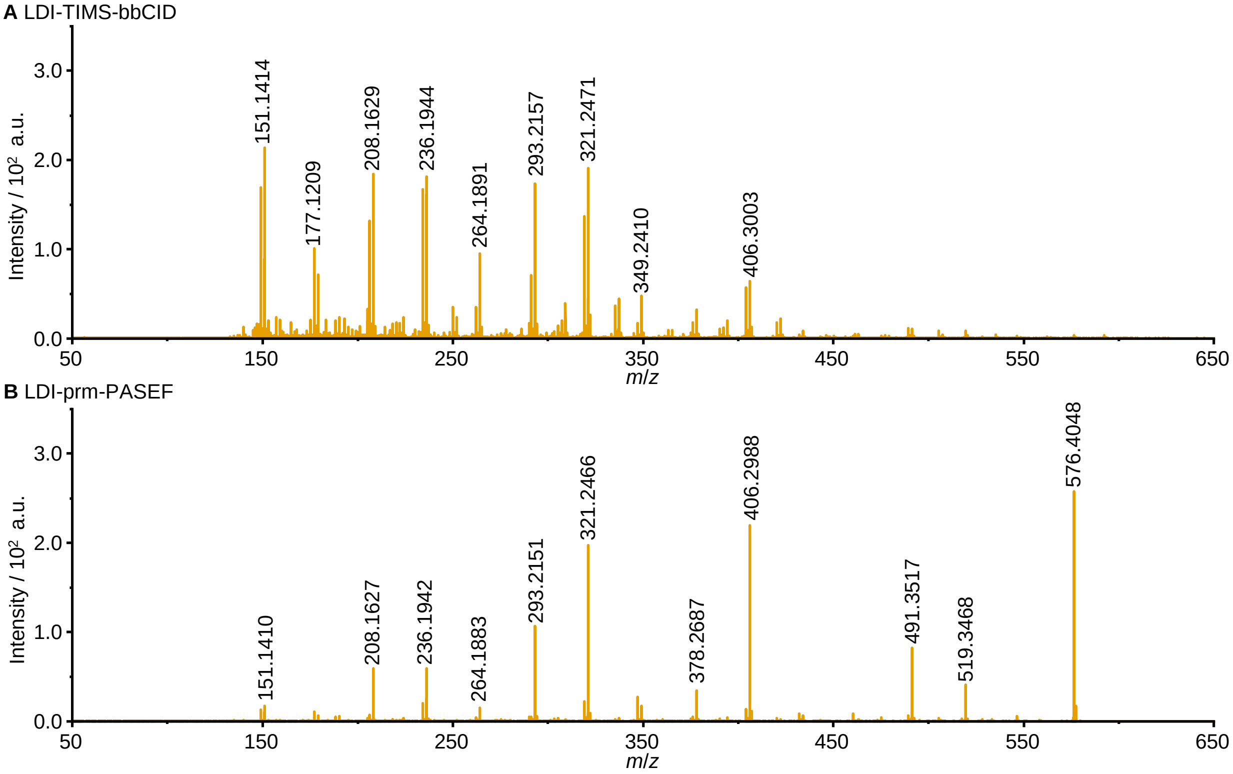

Figure S6.1. LDI-TIMS-MS/MS spectra of the electrochemically formed heptamer (*m/z* 576.4051) in the mobility range 1.069-1.113 V s cm^{-2} recorded using **A**: LDI-TIMS-bbCID at a collision energy of 70 eV and **B**: LDI-prm-PASEF at a collision energy determined by the parameters set in table 1 (see main manuscript).

S7 Proposed oligomerization mechanisms

Based on the acquired information on the formed Ala-*N*-CA oligomers, an oligomerization mechanism for the hydrolytically-induced oligomerization is proposed and the existing electrochemical oligomerization mechanism[3] is refined.

Hydrolytic oligomerization:

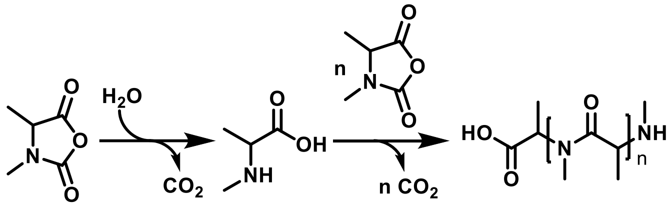

Ring opening reactions of anhydrides using oxynucleophiles such as water or alcohols are known side reactions of reactions involving anhydridic species which proceed at room temperature either with or without a catalyst added to the system.[4–6] In general, electrolyte additives for lithium ion batteries are supposed to be stable towards synthetic derivatization reactions and environmental impacts, but electrochemically instable, so they efficiently decompose into products that can form a stable and flexible SEI.[7] Since the synthesized title compound has an anhydride moiety, it is prone to hydrolytic cleavage as well. The hydrolytic decomposition of the *N*-CA structure leads to the ring-opened amino acid which can undergo addition to another *N*-CA molecule forming the hydrolytic polymer discussed in section S5.

Electrochemical oligomerization:

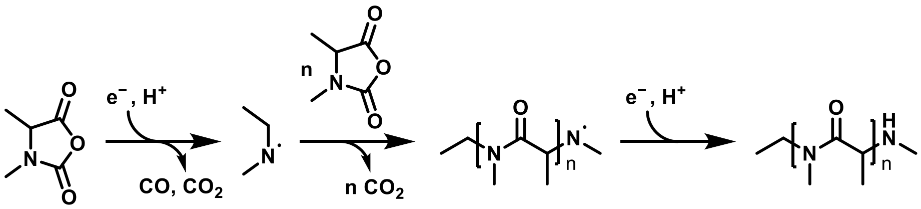

Some film-forming electrolyte additives are designed to release CO_2 upon reduction. Carbonate moieties are prone to release CO_2 after electrochemical reduction through firstly the cleavage of the C–O bond and subsequent release of CO_2.[8] In the case of *N*-CAs, both CO_2 and CO products could be detected via gas analysis,[3] which carbonate electrolytes are prone to in general as well,[9] corroborating the proposed mechanism.

Release of the two gases leads to formation of an amino radical which can undergo radical addition to another *N*-CA molecule forming an amide in the process. Oligomerization reactions lead to the formation of the detected electrochemical Ala-*N*-CA oligomer.

S8 LDI-MSI analysis

Imaging analysis was performed in triplicates to ensure the reliability and reproducibility of the acquired data. The mass spectrometric images showing the distribution of differently sized electrochemically formed Ala-*N*-CA oligomers on electrodes 2 and 3 are depicted in figure S8.1. The corresponding images acquired by analysis of electrode 1 are shown and discussed in the main manuscript. Similar to the electrode discussed in the main manuscript, the small oligomers show a more homogenous distribution on the electrode surfaces while the distribution becomes more focused on the edges of the electrodes for larger oligomers. A general decrease of signal intensities is observed in the lower left-hand side of electrode 2. This trend is observed for all signals and can be attributed to a detachment of the electrode from the sample slide in this area, causing the electrode surface to move out of the laser's focal plane. For the rest of the electrode, however, the laser focus was correct and the same trends are observed as for the two other electrodes.

In addition to the laser focus, the effect of ion suppression has to be considered during data evaluation. Overlying layers of other transformation products and electrolyte components might affect the ionization efficiency of the compounds of interest and create artefacts in the obtained images. Figure S8.2B shows the mass spectrometric image of the electrochemical Ala-*N*-CA hexamer while the distribution of the organophosphate with *m*/*z* 281.0165 discussed in section S3 is depicted in Figure S8.2C. For better comparability, an overlay image of the hexamer (red) and the organophosphate (blue) is shown in figure S8.2D. The two signals are inversely distributed. In areas of high signal intensity for the hexamer, small intensities are detected for the organophosphate and vice versa. This could indicate a suppression of the oligomer signal by overlying organophosphate. However, this suppression is not observed for the dimer (see Figure S8.2A) whose distribution shows little correlation with that of the organophosphate. While ion suppression cannot be completely ruled out to affect the signal distribution of the oligomers, a real difference in oligomer size in different areas of the electrode seems to exist.

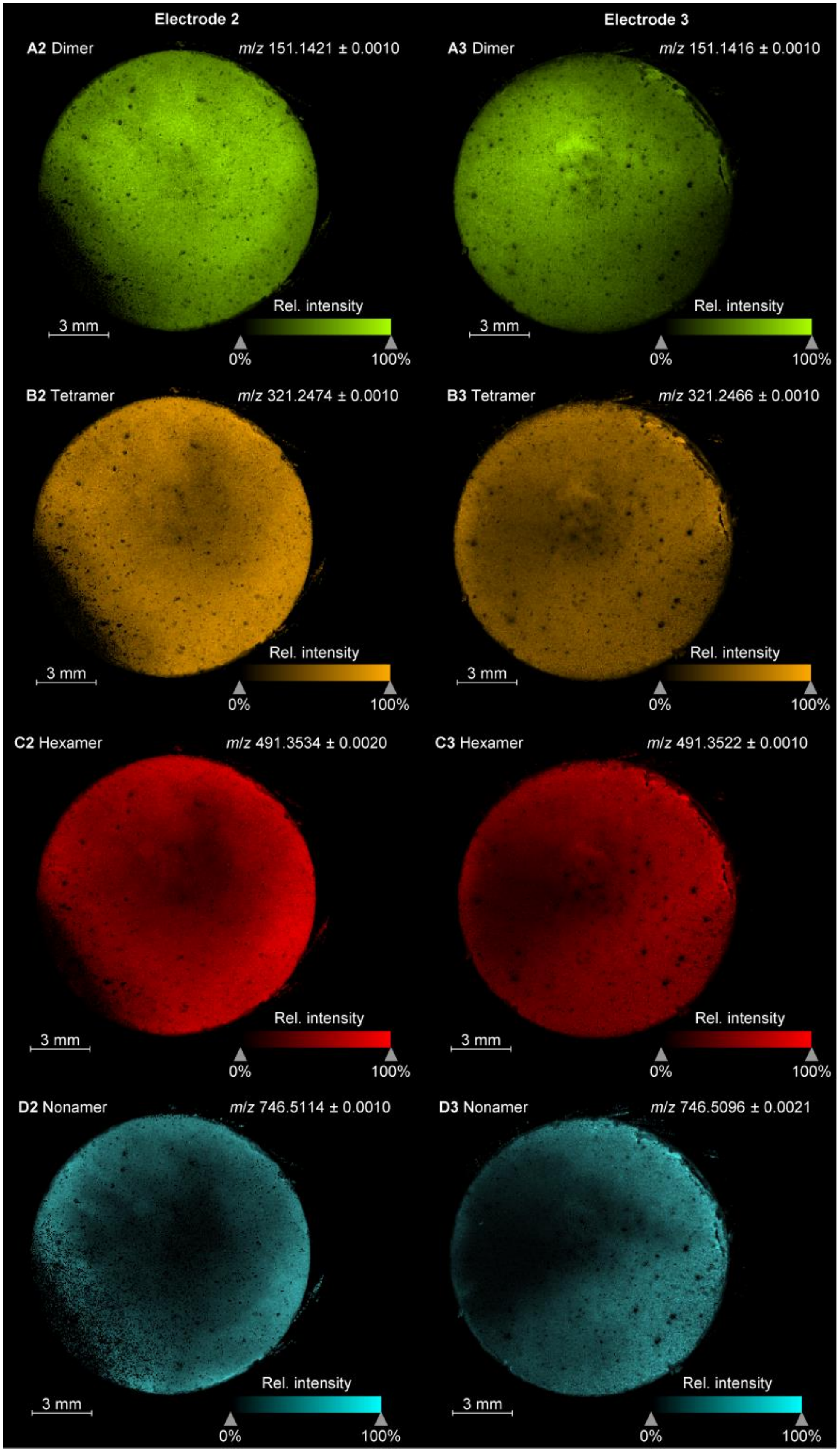

Figure S8.1. Mass spectrometric images of electrochemical oligomers on electrodes 2 and 3 from triplicate analysis. Images of electrode 1 are shown in the main manuscript.

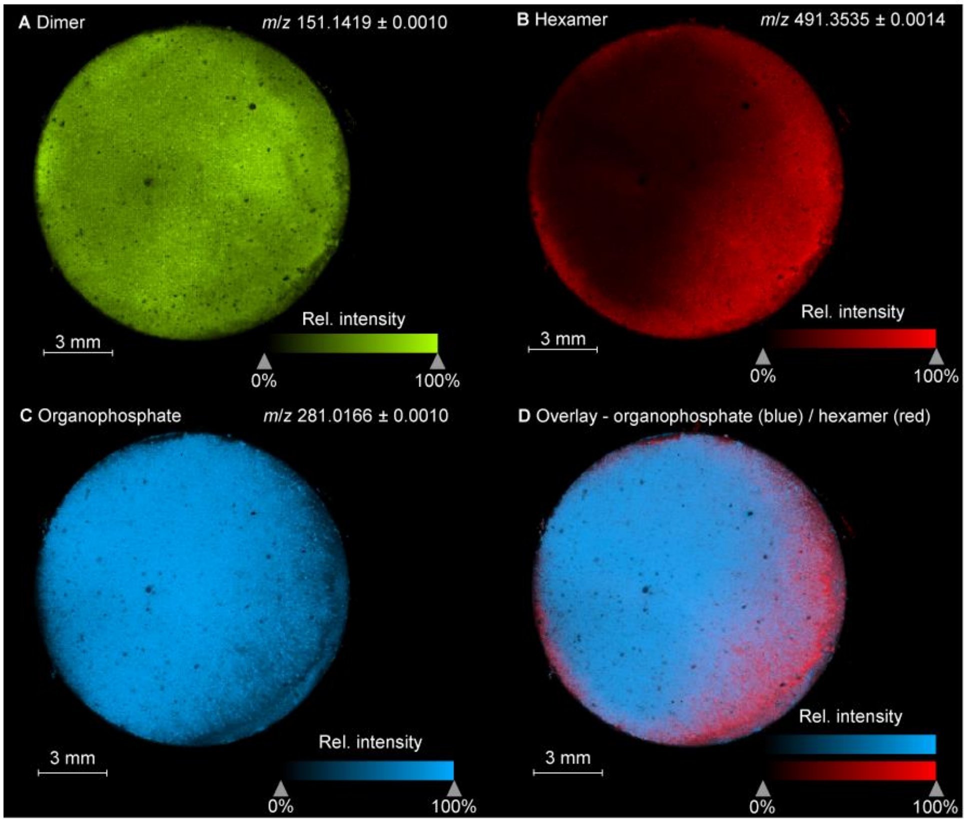

Figure S8.2. Mass spectrometric images of **A**: the electrochemical Ala-*N*-CA dimer on electrode 1, **B**: the electrochemical Ala-*N*-CA hexamer on electrode 1, **C**: the organophosphate on electrode 1 and **D**: an overlay of the electrochemical Ala-*N*-CA hexamer (red) and the organophosphate (blue) on electrode 1.

S9 Preparation of 3,4-dimethyloxazolidine-2,5-dione (Ala-*N*-CA)

Unless otherwise noted, all reactions were carried out in oven-dried glassware under an atmosphere of argon using standard Schlenk technique. Solvents were either used from a solvent purification system (HPLC grade) and dried via either an alumina or molecular sieves column under a positive argon pressure or purchased from Acros Organics (Geel, Belgium), Sigma-Aldrich (Steinheim, Germany) or Carl Roth (Karsruhe, Germany) and stored over molecular sieves.

Starting materials that were not synthesized in our laboratory, were obtained from commercial suppliers and used as received, unless otherwise noted.

Products were purified by flash column chromatography on Acros Organics silica gel (35–70 mesh). Suitable solvent mixtures for separation were identified by thin-layer chromatography (TLC) analysis on silica gel 60 F254 aluminum plates from Merck. TLC plates were visualized by irradiation with UV light (254 nm, 366 nm) and/or by staining in an alkaline $KMnO_4$ solution followed by heating.

NMR spectra were recorded on a Bruker Avance II 300 MHz, Bruker Avance II 400 MHz, Agilent DD2 500 MHz or on an Agilent DD2 600 MHz spectrometer. Chemical shifts (δ) are given in ppm. The residual solvent signals were used as references for ^{1}H and ^{13}C NMR spectra ($CDCl_3$: δ_H = 7.26 ppm, δ_C = 77.16 ppm; CD_2Cl_2: δ_H = 5.32 ppm, δ_C = 53.84 ppm). The multiplicities of the signals are described with standard abbreviations (s (singlet), d (doublet), t (triplet), q (quartet), p (pentet), hept (heptet) and m (multiplet)). Coupling constants (*J*) are quoted in Hz.

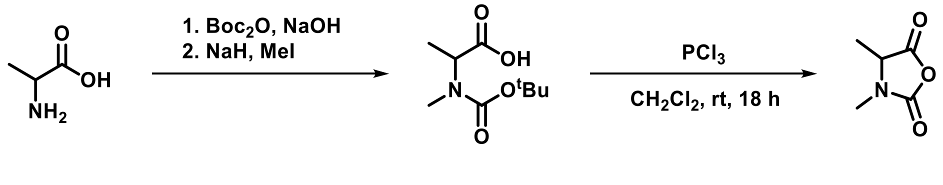

3,4-Dimethyloxazolidine-2,5-dione was synthesized following a literature procedure by Viallefont.[10] The title compound was prepared from the according amino acid L-alanine.

N-($Tert$-butoxycarbonyl)-N-methylalanine

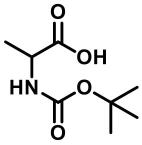

The amino acid was protected according to a modified literature procedure.[11,12] L-Alanine (891 mg, 10.0 mmol, 1.0 eq.) was dissolved in aq. NaOH (1 M, 10 mL). Then, di-*tert*-butyldicarbonate (2.62 g, 12.0 mmol, 1.2 eq.) was added and the reaction mixture was stirred 18 h at 23 °C. Subsequently, the mixture was acidified to pH 2 with aq. HCl (1 M), extracted with EtOAc, washed with brine and dried over $MgSO_4$. The solvent was removed yielding the product as an off-white solid (1.88 g, quant.). The product was used for the following step without further purification.

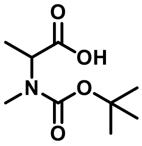

Methylation of the Boc-protected amino acid was carried out according to previously reported literature.[13] The solid (1.89 g, 10.0 mmol, 1.0 eq.) was dissolved in THF (60 mL) and the mixture was cooled to 0 °C. Sodium hydride (2.50 g, 100 mmol, 10 eq.) was added portionwise at that temperature. After stirring for 1 h at 0 °C, methyl iodide (3.88 mL, 100 mmol, 10 eq.) was added slowly. The reaction mixture was warmed to room temperature (RT) and stirred for 18 h. Subsequently, a few drops of H_2O were added to quench the reaction, EtOAc was added and all volatiles removed under reduced pressure. The remaining mixture was acidified to pH 2 und extracted with EtOAc. Drying over $MgSO_4$ and removing of solvent under reduced pressure yielded the product as a yellow oil (1.99 g, 9.80 mmol, 98%).

^{1}H NMR (400 MHz, $CDCl_3$) δ 11.30 (s, 1H), 4.94 – 4.36 (m, 1H), 2.85 (d, J = 13.2 Hz, 3H), 1.56 – 1.34 (m, 12H).

The NMR data is in accordance with reported values.[13].

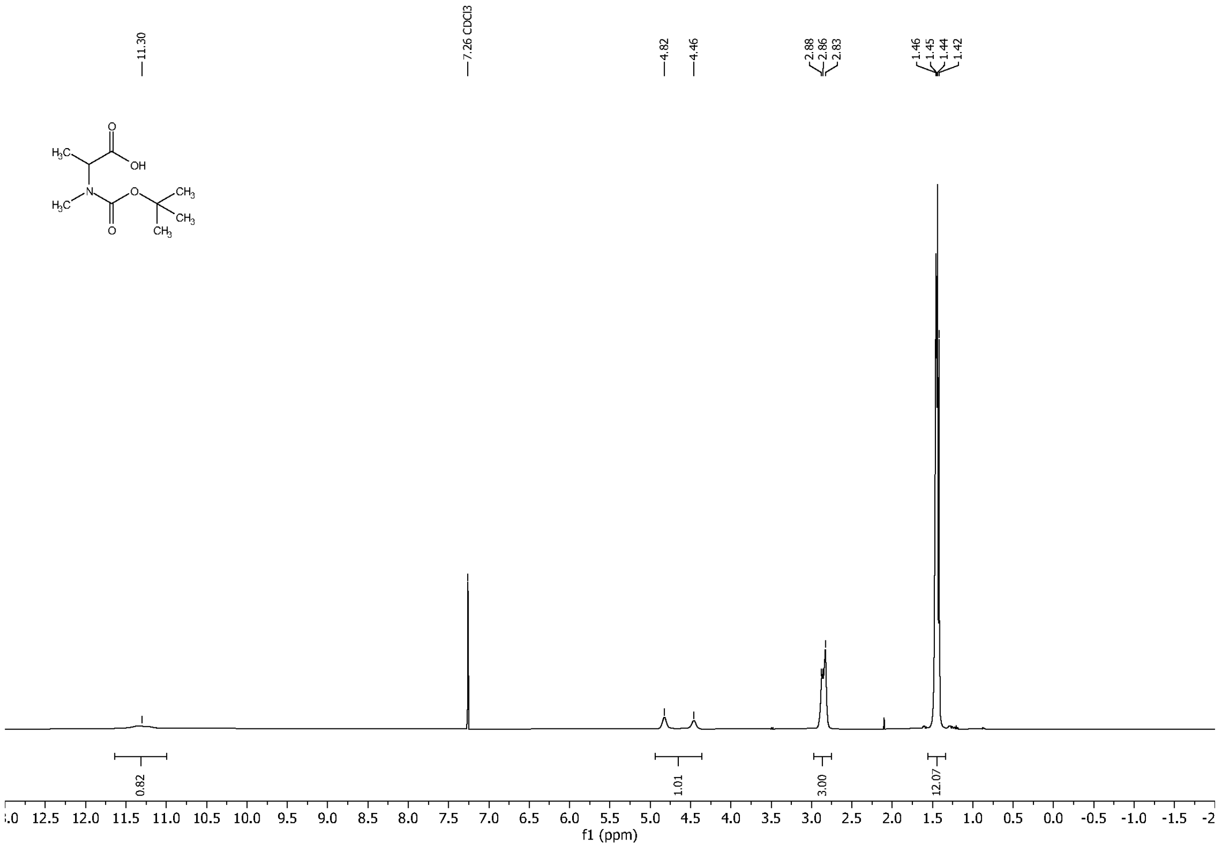

Figure S9.1. ^{1}H NMR spectrum of *N*-(*tert*-butoxycarbonyl)-*N*-methylalanine. in CD_2Cl_2.

3,4-Dimethyloxazolidine-2,5-dione

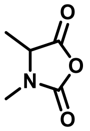

The title compound was prepared according to a modified literature procedure.[10] *N*-(*Tert*-butoxycarbonyl)-*N*-methylalanine (1.00 g, 4.90 mmol, 1.0 eq.) was dissolved in CH_2Cl_2 (15 mL) and cooled to 0 °C. Phosphorus trichloride (812 mg, 5.90 mmol, 1.2 eq.) was added dropwise and the reaction mixture was slowly warmed to RT. The mixture was stirred for 1 h at that temperature and subsequently, all volatiles were removed. The remaining solid was washed with cold CH_2Cl_2 and recrystallized from CH_2Cl_2 and hexane. The product was isolated as a colorless solid (580 mg, 4.50 mmol, 92%) and stored in an argon-filled glovebox.

^{1}H NMR (400 MHz, CD_2Cl_2) δ 4.18 (q, J = 7.0 Hz, 1H), 2.94 (s, 3H), 1.50 (d, *J* = 7.0 Hz, 3H).

^{13}C NMR (101 MHz, CD_2Cl_2) δ 170.0, 151.9, 57.3, 28.5, 15.2.

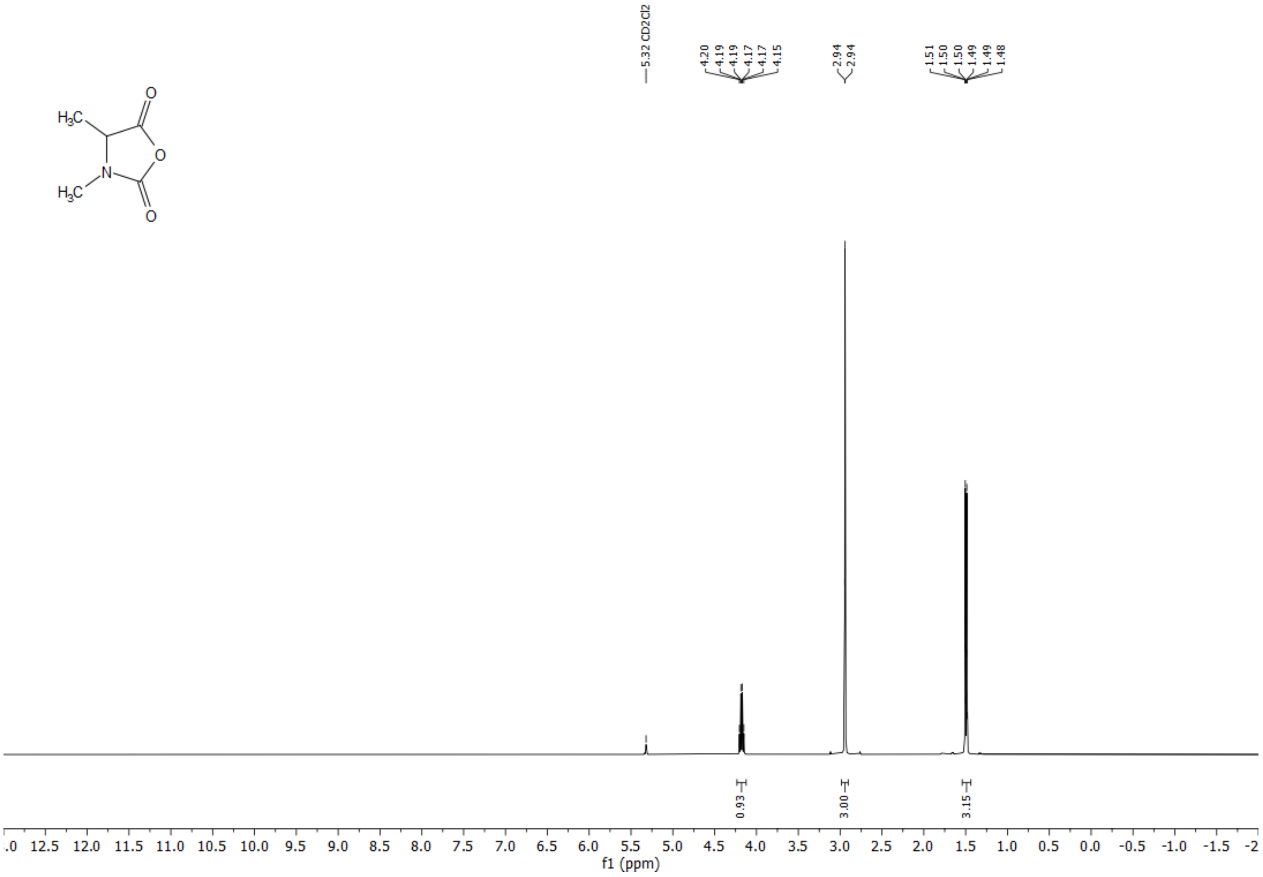

Figure S9.2. ^{1}H NMR spectrum of 3,4-dimethyloxazolidine-2,5-dione (Ala-*N*-CA) in CD_2Cl_2.

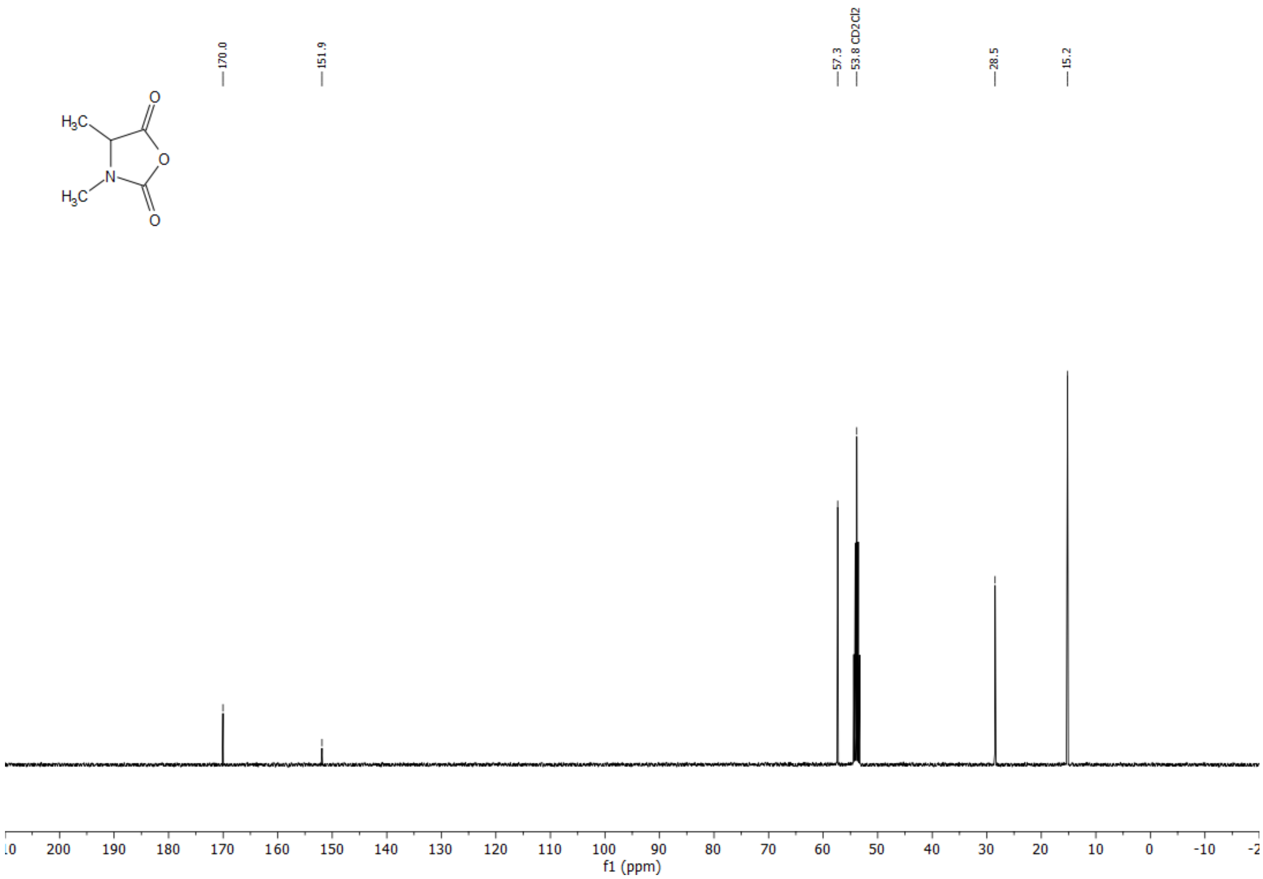

Figure S9.3. ^{13}C NMR spectrum of 3,4-dimethyloxazolidine-2,5-dione (Ala-*N*-CA) in CD_2Cl_2.

References

1. Henschel, J., Schwarz, J.L., Glorius, F., Winter, M., and Nowak, S. (2019). Further insights into structural diversity of phosphorus-based decomposition products in lithium ion battery electrolytes via liquid chromatographic techniques hyphenated to ion trap-time-of-flight mass spectrometry. Anal. Chem. *91*, 3980–3988. 10.1021/acs.analchem.8b05229.

2. Henschel, J., Peschel, C., Klein, S., Horsthemke, F., Winter, M., and Nowak, S. (2020). Clarification of Decomposition Pathways in a State-of-the-Art Lithium Ion Battery Electrolyte through 13C-Labeling of Electrolyte Components. Angew. Chem. Int. Ed. *59*, 6128–6137. 10.1002/anie.202000727.

3. Schmiegel, J.-P., Nölle, R., Henschel, J., Quach, L., Nowak, S., Winter, M., Glorius, F., and Placke, T. (2021). Case study of N-carboxyanhydrides in silicon-based lithium ion cells as a guideline for systematic electrolyte additive research. Cell Rep. Phys. Sci. *2*, 100327. 10.1016/j.xcrp.2021.100327.

4. Vedejs, E., and Daugulis, O. (1996). Dual activation in the esterification of hindered alcohols with anhydrides using MgBr2 and a tertiary amine. J. Org. Chem. *61*, 5702–5703. 10.1021/jo9609485.

5. Otera, J., and Nishikido, J. (2009). Esterification: Methods, Reactions, and Applications (Wiley).

6. Baruah, A.M., Karmakar, A., and Baruah, J.B. (2007). Hydrolytic ring opening reactions of anhydrides for first row transition metal dicarboxylate complexes. Polyhedron *26*, 4518–4524. 10.1016/j.poly.2007.06.028.

7. Ming, J., Cao, Z., Wu, Y., Wahyudi, W., Wang, W., Guo, X., Cavallo, L., Hwang, J.-Y., Shamim, A., Li, L.-J., et al. (2019). New Insight on the Role of Electrolyte Additives in Rechargeable Lithium Ion Batteries. ACS Energy Lett. *4*, 2613–2622. 10.1021/acsenergylett.9b01441.

8. Michan, A.L., Parimalam, B.S., Leskes, M., Kerber, R.N., Yoon, T., Grey, C.P., and Lucht, B.L. (2016). Fluoroethylene carbonate and vinylene carbonate reduction: Understanding lithium-ion battery electrolyte additives and solid electrolyte

interphase formation. Chem. Mater. *28*, 8149–8159. 10.1021/acs.chemmater.6b02282.

9. Shkrob, I.A., Zhu, Y., Marin, T.W., and Abraham, D. (2013). Reduction of carbonate electrolytes and the formation of solid-electrolyte interface (SEI) in lithium-ion batteries. 1. Spectroscopic observations of radical intermediates generated in one-electron reduction of carbonates. J. Phys. Chem. C *117*, 19255–19269. 10.1021/jp406274e.

10. Akssira, M., Boumzebra, M., Kasmi, H., Dahdouh, A., Roumestant, M.-L., and Viallefont, P. (1994). New routes to 1,4- benzodiazepin-2,5-diones. Tetrahedron *50*, 9051–9060. 10.1016/S0040-4020(01)85371-0.

11. Scharinger, F., Pálvölgyi, Á.M., Zeindlhofer, V., Schnürch, M., Schröder, C., and Bica-Schröder, K. (2020). Counterion Enhanced Organocatalysis: A Novel Approach for the Asymmetric Transfer Hydrogenation of Enones. ChemCatChem *12*, 3776–3782. 10.1002/cctc.202000414.

12. Chankeshwara, S. V., and Chakraborti, A.K. (2006). Catalyst-free chemoselective N-tert-butyloxycarbonylation of amines in water. Org. Lett. *8*, 3259–3262. 10.1021/ol0611191.

13. Loscher, S., and Schobert, R. (2013). Total synthesis and absolute configuration of epicoccamide D, a naturally occurring mannosylated 3-acyltetramic acid. Chem. - A Eur. J. *19*, 10619–10624. 10.1002/chem.201301914.
